# Development of Maize Hybrids With Enhanced Vitamin-E, Vitamin-A, Lysine, and Tryptophan Through Molecular Breeding

**DOI:** 10.3389/fpls.2021.659381

**Published:** 2021-07-21

**Authors:** Abhijit K. Das, Munegowda M. Gowda, Vignesh Muthusamy, Rajkumar U. Zunjare, Hema S. Chauhan, Aanchal Baveja, Vinay Bhatt, Gulab Chand, Jayant S. Bhat, Satish K. Guleria, Supradip Saha, Hari S. Gupta, Firoz Hossain

**Affiliations:** ^1^Division of Genetics, ICAR-Indian Agricultural Research Institute, New Delhi, India; ^2^Division of Genetics, IARI-Regional Research Centre, Dharwad, India; ^3^Plant Breeding, CSK Himachal Pradesh Krishi Vishvavidyalaya, Bajaura, India

**Keywords:** maize, biofortication, nutrition, marker-assist selection, hybrid, DUS traits

## Abstract

Malnutrition is a widespread problem that affects human health, society, and the economy. Traditional maize that serves as an important source of human nutrition is deficient in vitamin-E, vitamin-A, lysine, and tryptophan. Here, favorable alleles of *vte4* (α*-tocopherol methyl transferase*), *crtRB1* (β*-carotene hydroxylase*), *lcyE* (*lycopene* ε*-cyclase*), and *o2* (*opaque2*) genes were combined in parental lines of four popular hybrids using marker-assisted selection (MAS). BC_1_F_1_, BC_2_F_1_, and BC_2_F_2_ populations were genotyped using gene-based markers of *vte4, crtRB1, lcyE*, and *o2*. Background selection using 81–103 simple sequence repeats (SSRs) markers led to the recovery of recurrent parent genome (RPG) up to 95.45%. Alpha (α)-tocopherol was significantly enhanced among introgressed progenies (16.13 μg/g) as compared to original inbreds (7.90 μg/g). Provitamin-A (proA) (10.42 μg/g), lysine (0.352%), and tryptophan (0.086%) were also high in the introgressed progenies. The reconstituted hybrids showed a 2-fold enhancement in α-tocopherol (16.83 μg/g) over original hybrids (8.06 μg/g). Improved hybrids also possessed high proA (11.48 μg/g), lysine (0.367%), and tryptophan (0.084%) when compared with traditional hybrids. The reconstituted hybrids recorded the mean grain yield of 8,066 kg/ha, which was *at par* with original hybrids (mean: 7,846 kg/ha). The MAS-derived genotypes resembled their corresponding original hybrids for the majority of agronomic and yield-related traits, besides characteristics related to distinctness, uniformity, and stability (DUS). This is the first report for the development of maize with enhanced vitamin-E, vitamin-A, lysine, and tryptophan.

## Introduction

Malnutrition has become one of the alarming health problems leading to lower work efficiency and socio-economic losses worldwide (Allard, 1999). It affects two billion people especially in the developing countries (Arun et al., 2014). Malnutrition has been accounted for nearly 45% of deaths among children under the age of five (Azmach et al., 2013). Malnutrition contributes to loss in 11% gross domestic products (GDPs) in Asia and Africa, and in total, it could cost society up to US$3.5 trillion per year (International Food Policy Research Institute, 2018). Mild to moderate forms of micronutrient (vitamins and minerals) deficiency can severely affect human health and lead to mental impairment, thereby resulting in lower productivity in humans (Bouis, 2018). Global leaders have set the “Sustainable Development Goals” (SDGs) at United Nations in 2015 to alleviate poverty and hunger by 2030. It is estimated that with $1 investment in a proven nutrition programme, a benefit worth of $16 is achieved (International Food Policy Research Institute, 2017). Though various avenues like “food fortification,” “medical supplementation,” and “dietary diversification” are available as strategies to alleviate malnutrition, the development of biofortified crops with enhanced micronutrients still remains the most cost-effective way to accomplish these SDGs (Andersson et al., 2017).

Maize is a primary source of energy and food for hundreds of millions of people globally (Shiferaw et al., 2011). However, traditional maize possesses low vitamin-E (6–8 μg/g), vitamin-A (1–2 μg/g), lysine (0.15–0.20%), and tryptophan (0.03–0.04%), which play a crucial role in human metabolism (Zunjare et al., 2017, 2018a; Hossain et al., 2018; Das et al., 2019b). Vitamin-E quenches free radicals in the cell membrane, thereby protecting the polyunsaturated fatty acids (PUFA) from damage; besides, it also serves as an essential micronutrient for the proper functioning of the reproduction system. It further protects from cardiovascular disease, Alzheimer's disease, neurological disorder, and many age-related degenerations (Chander et al., 2008). Vitamin-E deficiency (VED) has been observed more in premature infants and elderly people (Das et al., 2018). It is estimated that over 20% of the examined people both in developed and in developing countries have suboptimal plasma alpha (α)-tocopherol, the most active form of vitamin-E (Li et al., 2012). Vitamin-A deficiency (VAD) is also a major health problem worldwide, and it causes visual impairment and results in low resistance to infectious diseases (Black et al., 2008). Vitamin-A deficiency affects about 20 million pregnant women, and one-third of them are clinically night-blind. It also affects 250 million children and accounts for increased childhood mortality and disease (WHO, 2009). Besides vitamins, lysine and tryptophan serve as essential amino acids for protein synthesis inside the human body, and deficiency of which leads to the most common symptoms such as loss of appetite, depression, delayed growth, and anxiety in children (Nuss and Tanumihardjo, 2010). Among nutritional disorders, the highest number of death occurs due to protein-related deficiencies worldwide (Bain et al., 2013; Hossain et al., 2019). Health benefits of quality protein maize (QPM) in human and the growth of poultry birds and pigs have also been reported (Gunaratna et al., 2010; Panda et al., 2014). Provitamin-A (proA)-rich biofortified maize was effective in reducing VAD in children (Gannon et al., 2014). Further, chickens fed with proA-rich maize kernel showed more redness in meat color and produced eggs with a higher amount of proA (Heying et al., 2014; Moreno et al., 2016; Odunitan-Wayas et al., 2016; Sowa et al., 2017). Over the last decade, efforts have been made for vitamin-E biofortification in different crops (Mene-Saffrane and Pellaud, 2017). In Arabidopsis, the introduction of γ-tocopherol methyltransferase (γ-TMT) resulted in an increased accumulation of α-tocopherol over γ-tocopherol in seeds (Shintani and DellaPenna, 1998; Mene-Saffrane and Pellaud, 2017). Similar results were also witnessed in other crops, including soybean (Arun et al., 2014) and lettuce (Cho et al., 2005).

Thus, the development of maize hybrids rich in these nutrients holds an immense promise to alleviate malnutrition in a holistic way. Maize mutants with a favorable allele of *vte4* gene (α*-tocopherol methyl transferase*) accumulating 2–3 fold more vitamin-E have been identified (Li et al., 2012; Das et al., 2018). The natural variants of *crtRB1* (β*-carotene hydroxylase*) and *lcyE* (*lycopene* ε*-cyclase*) that enhance vitamin-A by 2–10 fold are also available in maize germplasm (Harjes et al., 2008; Yan et al., 2010). The recessive *opaque2* (*o2*) mutant allele increases lysine and tryptophan by 2-fold (Mertz et al., 1964; Hossain et al., 2018). Several QPM hybrids with *o2* gene and proA-rich maize hybrids with *crtRB1* and *lcyE* genes have been released worldwide (Cabrera-Soto et al., 2018; Hossain et al., 2019; Prasanna et al., 2020). In India, ICAR-Indian Agricultural Research Institute (ICAR-IARI), New Delhi, have developed (first of its kind) three proA-rich QPM hybrids for commercial cultivation (Muthusamy et al., 2014; Zunjare et al., 2018a). However, these hybrids still lack vitamin-E, which is essential for proper metabolism in humans (Das et al., 2019b). Thus, the development of maize hybrids rich in vitamin-E, vitamin-A, and essential amino acids holds immense significance in alleviating malnutrition in a sustainable and cost-effective way. Marker-assisted selection (MAS) is the ideal approach to combine desirable allele of multiple genes in maize breeding programme for the release of commercial hybrids (Yadava et al., 2018; Prasanna et al., 2020). Hence, the current study was undertaken to (i) combine favorable alleles of *vte4, crtRB1, lcyE*, and *o2* genes into the parental lines of four released hybrids using MAS, (ii) evaluate the introgressed inbred lines and reconstituted hybrids for nutritional quality traits, and (iii) assess the genotypes for agronomic and yield-related traits. This is the first report of combining vitamin-E with vitamin-A, lysine, and tryptophan in a maize hybrid.

## Materials and Methods

### Plant Materials

Four QPM inbreds improved for proA (HKI161-VA, HKI163-VA, HKI193-1-VA, and HKI193-2-VA) by Zunjare et al. (2018a) were selected for the enrichment of α-tocopherol (vitamin-E). These four inbreds are the parents of four proA-rich QPM hybrids (HQPM1-VA, HQPM4-VA, HQPM5-VA, and HQPM7-VA). The details of these hybrids are presented in Supplementary Table 1. The parental lines possess favorable alleles of *o2, crtRB1*, and *lcyE* genes, and are higher in lysine, tryptophan, and proA. However, all the four inbreds had a lower level of α-tocopherol. The donor inbred (HP465-41) developed at International Maize and Wheat Improvement Center (CIMMYT), Mexico, under the HarvestPlus programme was used for introgression of the favorable allele of *vte4* into these parental lines. The donor line also possessed favorable alleles of *crtRB1* and *lcyE* genes. Pedigree of donor and recipient parents is given in Supplementary Table 2.

### Marker-Assisted Backcross Breeding (MABB) Scheme

Each of the recipient parents was crossed as female with donor parent as male during the winter season of 2014–2015 at Winter Nursery Center (WNC) of ICAR-Indian Institute of Maize Research (ICAR-IIMR), Hyderabad (17°19′N, 78°24′E, 542.6 MSL) (N, north; E, east; MSL, mean sea level). F_1_ plants were raised during the rainy season (2015) at ICAR-IARI, New Delhi (28°08′N, 77°12′E, 229 MSL), and tested for hybridity using *vte4* and *o2* specific markers. True heterozygotes were backcrossed as male with the respective recurrent parents. BC_1_F_1_ progenies were grown at WNC, Hyderabad, during the winter season of 2015–2016, and foreground positive (heterozygous for *vte4* and *o2*; homozygous for *crtRB1* and *lcyE* favorable allele) individuals were tagged before flowering. Individuals with more similarity to recurrent parents and high recovery of recurrent parent genome (RPG) were further backcrossed with the respective recurrent parents. BC_2_F_1_ progenies were grown during the rainy season (2016) at ICAR-IARI, New Delhi, and foreground positive plants (heterozygous: *vte4* and *o2*; homozygous: *crtRB1* and *lcyE*) were selected. Genotypes with high RPG and phenotypic similarity were advanced to raise BC_2_F_2_ progenies at WNC, Hyderabad, during the winter season of 2016–2017. The segregants homozygous for all the four genes (*vte4, crtRB1, lcyE*, and *o2*) were identified, and plants with high RPG and phenotypic similarity were selected. The BC_2_F_3_ progenies were raised during the rainy season (2017) at ICAR-IARI, New Delhi, and advanced to BC_2_F_4_ progenies for the generation of crosses during the winter season at WNC, Hyderabad. The reconstituted hybrids were evaluated during the rainy season (2018) at multilocations.

### Isolation of DNA and Polymerase Chain Reaction Analysis

Genomic DNA (deoxy ribonucleic acid) was isolated from the leaf of young maize seedlings using the standard CTAB (cetyl trimethyl ammonium bromide) protocol (Murray and Thompson 1980). polymerase chain reaction (PCR) was performed using (i) *InDel7* (InDel: insertion–deletion) and *InDel118* markers for *vte4*, (ii) *3*′*TE-InDel* marker for *crtRB1* (TE: transposable element), (iii) *5*′*TE-InDel* marker for *lcyE*, and (iv) *phi057* (simple sequence repeat: SSR) for *o2* (Supplementary Table 3). The product size of favorable alleles for *InDel118* and *InDel7* was 373 and 160 bp, whereas that of unfavorable alleles was 491 and 167 bp, respectively. For *crtRB1-3*′*TE* and *lcyE-5*′*TE*, the amplified product for favorable alleles was 543 and 650 bp, respectively. The favorable allele of *phi057* in recurrent parents was of 165 bp, and the unfavorable donor allele was of 159 bp. For *vte4*, PCR was performed following the protocol of Li et al. (2012). Amplified product for *InDel118* was resolved using 2% agarose gel, and that for *InDel7* was resolved using 4% super fine resolution (SFR) agarose. For *3*′*TE-InDel of crtRB1*, PCR was performed as suggested by Yan et al. (2010), and 1.5% agarose gel was used to resolve the amplified products. For *5*′*TE-InDel of lcyE*, PCR was performed as suggested by Harjes et al. (2008), and products were resolved using 2% agarose gel. PCR amplified products of SSRs for o2 and background markers was resolved using 4% agarose as per PCR protocol as suggested by Hossain et al. (2018).

### Foreground Selection

Two insertion/deletions (*InDel7* and *InDel118*) at the promoter and 5′-UTR region of *vte4* gene were identified by Li et al. (2012). Donor parent had favorable haplotype (0/0: deletion at both locations), whereas all the four recipient parents possessed unfavorable allele (7/118: insertion at both locations) of *vte4*. Since *InDel7* and *InDel118* are in close proximity within the *vte4* gene, *InDel118* was sufficient for genotyping of *vte4* locus in backcross generations. Markers specific to *3*′*TE InDel* (in exon 6 and 3′-UTR) of *crtRB1* gene and *5*′*TE InDel* (in 5′-UTR) of *lcyE* gene were used to select for high proA. Gene-based SSR (*phi057*) was used as a foreground marker for *o2*. The detail of the markers along with location in the genome used for the foreground selection is presented in Supplementary Table 3. Foreground selection was accomplished for the identification of plants heterozygous for *vte4* and *o2* and homozygous for the favorable allele of *crtRB1* and *lcyE* in BC_1_F_1_ and BC_2_F_1_. In BC_2_F_2_ generation, plants homozygous for favorable allele all four genes were selected.

### Background Selection

A set of 285 SSRs distributed throughout the maize genome covering all the 10 chromosomes was selected from the maize genome database (www.maizegdb.org) (Supplementary Table 4). These selected SSRs were used to identify polymorphic markers between the respective recurrent and donor parents. Respective polymorphic SSRs set between recurrent and donor parents were used for the identification of genotypes with high RPG in BC_1_F_1_, BC_2_F_1_, and BC_2_F_2_.

### Evaluation of Introgressed Progenies

Fourteen BC_2_F_4_ progenies—four each from HKI161-VA and HKI193-1-VA; three each from HKI163-VA and HKI193-2-VA, along with the four recurrent parents—were grown in a randomized complete block design (RCBD) with two replications during the rainy season (2018) at ICAR-IARI Experimental Farm, New Delhi. Each entry was grown in a row of 3 m length, and plant-to-plant distance of 20 cm and row-to-row distance of 75 cm were maintained, and standard agronomic practices were followed to raise a good crop. In each row, 2–3 plants were self-pollinated to avoid xenia effects, and selfed seeds were used for the quality analysis. Grain yield, male and female flowering, plant and ear height, ear length, ear diameter, number of rows per ear, number of kernels per row, and 100 kernel weight were recorded from open pollinated plants. Further, all the 31 characteristics related to distinctness, uniformity, and stability (DUS) were also recorded in each of the introgressed lines and recurrent parent.

### Evaluation of Reconstituted Hybrids

The introgressed lines were used to reconstitute hybrid combinations representing three versions (I, II, III) in each of the four original hybrids during the winter season (2017–2018) (Supplementary Table 5). The reconstituted 12 hybrid combinations and their corresponding original four hybrids along with a commercial check, Pusa Vivek QPM9 Improved (PVQ9I), were evaluated at three locations, *viz*. Bajaura (32°2′N, 77°9′E, 1,090 MSL), Delhi (28°08′N, 77°12′E, 229 MSL), and Dharwad (15°21′N, 75°05′E, MSL: 750 MSL) during the rainy season of 2018. PVQ9I is a proA-rich QPM hybrid released and notified for cultivation during 2017 in India. These three locations belong to three different maize-growing zones of the country. While Bajaura is situated in Northern Hills Zone, Delhi and Dharwad belong to North Western Plains Zone and Peninsular Zone, respectively. Hybrids were evaluated in RCBD with two replications. In each entry, 2–3 plants were self-pollinated to avoid contamination by foreign pollens, and selfed seeds were used for nutritional quality analysis. Grain yield, male and female flowering, plant height, ear height, ear length, ear girth, number of rows per ear, number of kernels per row, and 100 kernel weight were recorded from the open-pollinated plants. Further, the hybrids were also characterized for all the 31 DUS traits.

### Biochemical Analysis

#### Estimation of Tocopherol

Seeds were stored at 4°C until extraction to avoid any degradation of quality traits. Extraction of tocopherol was performed following the protocol of Saha et al. (2013), but absolute ethanol was used instead of methanol. Twenty microliters of each sample were injected into the Dionex Ultimate 3000 UHPLC System (Ultra High Performance Liquid Chromatography; Thermo Scientific, Massachusetts, United States), and the fluorescence detector was used with an excitation wavelength of 290 nm and an emission wavelength of 325 nm to detect the peak. Reverse-phase column YMC-C_30_ (5 μm, 4.6 × 250mm; Waters Chromatography) was used. Methanol and TBME (tert-butyl methyl ether) in a ratio of 95:5 (v/v) were used as a mobile phase with a flow rate of 1 ml min^−1^. Different dilutions (50, 100, 500, and 1,000 ppm) of each of the tocopherol standards, *viz*. alpha (α)-, beta (β-), gamma (γ)-, and delta (δ)-tocopherol (SIGMA chemicals, United States), were used to prepare the standard curve, which was further used to determine the concentration of respective tocopherols in samples by the standard regression equation.

#### Estimation of β-Carotene and β-Cryptoxanthin

The protocol described by Kurilich and Juvik (1999) and Vignesh et al. (2012) was used for the extraction of β-carotene and β-cryptoxanthin, and quantification was performed using Dionex Ultimate 3000 UHPLC System. YMC carotenoid C_30_ column (5 μm, 4.6 × 250mm; YMC) was used to elute the sample that was detected with a diode array detector-3000 (RS) at 450nm. The mobile phase consisted of methanol:TBME (80:20, v/v) with a flow rate of 1 ml min^−1^. Concentration of β-carotene and β-cryptoxanthin was calculated from the standard regression curve prepared from different dilutions of corresponding standards (Sigma Aldrich, United States). Total β-carotene content and half of the β-cryptoxanthin content of each sample were used to calculate the proA concentration (Babu et al., 2013).

#### Estimation of Lysine and Tryptophan

Lysine and tryptophan contents were extracted following the protocol standardized by Sarika et al. (2018), and the Dionex Ultimate 3000 UHPLC system was used for estimation. The Acclaim^TM^ 120 C_18_ column (5 μm, 120 A°, and 4.6 × 150mm, Thermo Scientific) was used to elute the samples, and detection was done with a RS photodiode array detector (PDA) at 265 and 280 nm wavelength for lysine and tryptophan, respectively. Amino acid concentration of each sample was estimated from the standard regression curve prepared using external standards (AAS 18-5ML, Sigma Aldrich).

### Statistical Analysis

χ^2^-test for goodness of fit was performed using SPSS16 (SPSS Inc. Released 2007) to check the Mendelian segregation of genes in BC_1_F_1_, BC_2_F_1_, and BC_2_F_2_ generations. Simple sequence repeats-amplified fragments of foreground positive plants were scored as “A” to represent the recipient allele, “B” to represent the donor allele, and “H” for the heterozygote. Calculation of RPG recovery and graphical representation of background genome of selected individuals in backcross generations were done using Graphical Geno Types (GGT) version 3.0 (Van-Berloo, 1999). Windostat 8.5 software package was used for the calculation of analysis of variance (ANOVA) and standard error (SE) values for agronomic and biochemical data of improved inbreds and hybrid.

## Results

### Polymorphism for Targeted Genes

Screening of donor and recipient parents using *InDel118* and *InDel7* markers of *vte4* revealed the presence of favorable alleles in donor parent and unfavorable alleles in recurrent parents for both *InDels*. *InDel118* produced easily distinguishable alleles between recipient and donor parents (allele difference of 118 bp), compared to *InDel7* (allele size difference of 7 bp). Since *InDel118* and *InDel7* are closely linked and present within the same gene, the selection of *InDel118* could invariably select *InDel7* as well. Hence, only *InDel118* was used for foreground selection to introgress the favorable allele of *vte4*. For *o2* gene, the use of *phi057* primer pair had amplified favorable allele in each of the four recurrent parents and the unfavorable allele in the donor parent. Recipient and donor parents both amplified the favorable alleles of *crtRB1* and *lcyE* using *3*′*TE-InDel* and *5*′*TE-InDel*, respectively.

### Generation of F_1_ Hybrids and Test of Hybridity

The donor parent was crossed as male with each of the recurrent parents. All the F_1_s were heterozygote for *vte4* and *o2* genes and homozygous for the favorable allele of *crtRB1* and *lcyE*.

### Simple Sequence Repeat Polymorphism for Background Selection

A total of 285 markers were used for the polymorphism survey between the donor and recurrent parents. Parental polymorphism survey identified 103, 81, 89 and 81 polymorphic SSRs amounting to 36.14, 28.42, 31.22, and 28.42% of polymorphism between the donor (HP465-41) and HKI161-VA, HKI163-VA, HKI193-1-VA, and HKI193-2-VA, respectively (Supplementary Table 4). Number of polymorphic markers ranged from 4 to 15 per linkage group. Respective polymorphic markers were used to recover the background genome of the recipient parent in each of the crosses.

### Genotyping of Segregating Populations

#### BC_1_F_1_ Populations

Population size of BC_1_F_1_ ranged from 96 to 124 across four genetic backgrounds (Table 1). Genotyping of BC_1_F_1_ populations with *InDel118* identified 62, 53, 51, and 66 heterozygous individuals in HKI161-VA, HKI163-VA, HKI193-1-VA, and HKI193-2-VA, respectively. Chi-square for the goodness of fit showed the Mendelian segregation pattern (1:1) for *vte4* in each of the crosses (Table 1). *Vte4* heterozygote genotypes from each of the populations were further screened for *o2* homozygote (*o2o2*) using *phi057*. Thirty plants of HKI161-VA, 22 plants of HKI163-VA, 21 plants of HKI193-1-VA, and 24 plants of HKI193-2-VA were homozygous for favorable *o2* allele. In the case of HKI161-VA-, HKI163-VA-, and HKI193-1-VA-based populations, Mendelian segregation of 1:1 was observed, while in HKI-193-2-VA-based population it significantly deviated from the Mendelian segregation. These selected plants were screened for *crtRB1* and *lcyE* genes, and all were homozygous for the favorable alleles. Owing to cross-pollinated nature of maize, the genotyping for *crtRB1* and *lcyE* was carried out to avoid any cross-pollination (by wild-type plants grown elsewhere in the field) that may lead to heterozygosity and later on fixation of an unfavorable allele in the progeny. Phenotypic selection for individuals that were heterozygous for *vte4* and homozygous for *o2, crtRB1*, and *lcyE* reduced the number to 10 for each family. Recurrent parent genome among the selected segregants varied from 61.70 to 77.20% across the populations (Table 2). Based on the plant, ear, and grain characteristics, two selected ears each in HKI161-VA (HKI161-VA-19: 72.85% and HKI161-VA-57: 76.10%), HKI163-VA (HKI163-VA-8: 77.20% and HKI-VA-38: 75.65%), and HKI193-1 (HKI193-1-VA-10: 75.00% and HKI-VA-20: 75.30%) and one in HKI193-2 (HKI-193-2-VA: 76.75%) were advanced to raise BC_2_F_1_ populations (Table 2).

**Table 1:** Segregation of *vte4* and *o2* genes in backcross generations.

**Cross**	**Stage**	***N***	***V^**+**^V^**+**^***	***V^**+**^V***	***VV***	***Chi square***	***p***	***N***	***Oo***	***oo***	***Chi square***	***p***
HKI161-VA × HP465-41	BC_1_F_1_	120	58	62	–	0.13	0.72^ns^	62	32	30	0.07	0.79 ^ns^
	BC_2_F_1_	110	53	57		0.15	0.70^ns^	57	–	57	–	–
	BC_2_F_2_	96	26	45	25	0.40	0.82^ns^	25	–	25	–	–
HKI163-VA × HP465-41	BC_1_F_1_	112	59	53	–	0.32	0.57^ns^	53	31	22	1.53	0.21 ^ns^
	BC_2_F_1_	120	57	63		0.30	0.58^ns^	63	–	63	–	–
	BC_2_F_2_	116	28	59	29	0.05	0.97^ns^	29	–	29	–	–
HKI193-1-VA × HP465-41	BC_1_F_1_	98	47	51	–	0.16	0.69^ns^	51	30	21	1.59	0.20 ^ns^
	BC_2_F_1_	122	62	60		0.03	0.86^ns^	60	–	60	–	–
	BC_2_F_2_	120	33	55	32	0.85	0.65^ns^	32	–	32	–	–
HKI193-2-VA × HP465-41	BC_1_F_1_	124	58	66	–	0.52	0.47^ns^	66	42	24	4.91	0.03^*^
	BC_2_F_1_	100	54	46		0.64	0.42^ns^	46	–	46	–	–
	BC_2_F_2_	112	26	58	28	0.21	0.90^ns^	28	–	28	–	–

*ns, Non-significant; N, number of plants genotyped; V^+^, unfavorable allele of vte4; V, favorable allele of vte4; O, unfavorable allele of opaque2; o, favorable allele of opaque2*.

* *Significant at 5% level of significance*.

**Table 2:** Recurrent parent genome (RPG) recovery in the backcross progenies.

**Cross**	**Generation**	**Genotype advanced**	**RPG Recovery (%)**	**Range of RPG (%)**
HKI161-VA × HP465-41	BC_1_F_1_	HKI161-VA-19	72.85	70.90–77.15
		HKI161-VA-57	76.10	
	BC_2_F_1_	HKI161-VA-19-4	88.45	82.25–90.60
		HKI161-VA-57-5	88.70	
	BC_2_F_2_	HKI161-VA-19-4-18	91.10	90.9–95.45
		HKI161-VA-19-4-54	91.10	
		HKI161-VA-19-4-60	95.45	
		HKI161-VA-57-5-4	91.70	
HKI163-VA × HP465-41	BC_1_F_1_	HKI163-VA-8	77.20	61.70–77.20
		HKI163-VA-38	75.65	
	BC_2_F_1_	HKI163-VA-8-52	89.1	79.95–89.10
		HKI163-VA-8-82	87.3	
		HKI163-VA-38-5	86.6	
	BC_2_F_2_	HKI163-VA-8-52-26	91.15	85.95–91.15
		HKI163-VA-8-82-18	90.20	
		HKI163-VA-38-5-41	87.70	
HKI193-1-VA × HP465-41	BC_1_F_1_	HKI193-1-VA-10	75.00	64.85–76.25
		HKI193-1-VA-20	75.30	
	BC_2_F_1_	HKI193-1-VA-10-5	87.35	80.40–87.95
		HKI193-1-VA-20-37	87.95	
	BC_2_F_2_	HKI193-1-VA-10-5-19	91.45	84.45–93.35
		HKI193-1-VA-10-5-36	84.45	
		HKI193-1-VA-20-37-30	90.80	
		HKI193-1-VA-20-37-64	93.35	
HKI1193-2-VA × HP465-41	BC_1_F_1_	HKI193-2-VA-1	76.75	62.70–76.75
	BC_2_F_1_	HKI193-2-VA-1-93	86.25	81.00–86.95
		HKI193-2-VA-1-94	86.25	
	BC_2_F_2_	HKI193-2-VA-1-93-45	85.50	85.50–92.25
		HKI193-2-VA-1-94-70	88.75	
		HKI193-2-VA-1-94-84	88.75	

#### BC_2_F_1_ Populations

The population size in BC_2_F_1_ varied from 100 to 122. Genotyping with *InDel118* revealed that 57, 63, 60, and 46 plants were heterozygotes in populations of HKI161-VA, HKI163-VA, HKI193-1-VA, and HKI193-2-VA, respectively (Table 1). The Chi-square (χ^2^) test for goodness of fit indicated the Mendelian segregation of 1:1. Each of the foreground positive plants (heterozygous for *vte4*) was screened for *o2, crtRB1*, and *lcyE*. As per expectation, all the plants heterozygous for *vte4* were homozygous for favorable alleles of the three genes. Phenotypic selection further reduced them to 10 individuals in populations of HKI161-VA, HKI163-VA, and HKI193-1-VA, while it reduced them to nine individuals for HKI193-2-VA population. Recurrent parent genome in these selected segregants ranged from 79.95 to 90.60% across populations (Table 2). Nine segregants, HKI161-VA-19-4 (88.45%), HKI161-VA-57-5 (88.70%), HKI163-VA-8-52 (89.1%), HKI163-VA-8-82 (87.3%), HKI163-VA-38-5 (86.6%), HKI193-1-VA-10-5 (87.35%), HKI193-1-VA-20-37 (87.95%), HKI193-2-VA-1-93 (86.25%), and HKI193-2-VA-1-94 (86.25%), were finally selected based on plant, ear and grain characteristics. These selected plants were selfed to raise BC_2_F_2_ populations.

#### BC_2_F_2_ Populations

**Figure 1:**
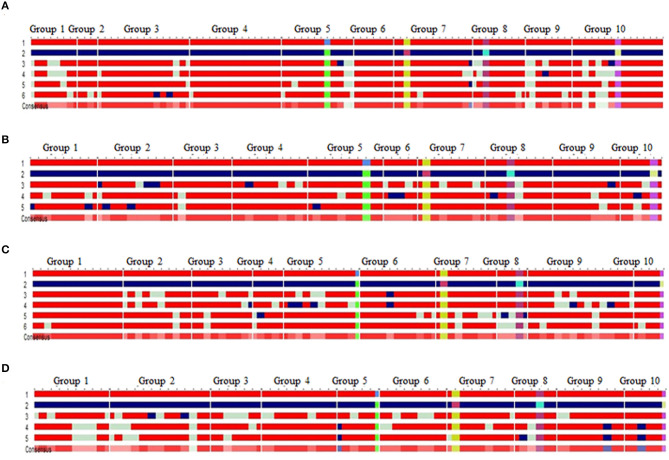
Background recovery of selected progenies in BC_2_F_2_ of the RP × DP [**(A)** HKI161-VA+VE, **(B)** HKI163-VA+VE, **(C)** HKI193-1-VA+VE, and **(D)** HKI193-2-VA+VE]; 1, recurrent parent (red); 2, donor parent (blue); light green color represent the locus in heterozygous condition; light red color in “consensus sequence” represents the locus having donor allele or heterozygosity, 3–5/6: introgressed progenies; favorable allele of *vte4* (green box), favorable allele of *phi057* (yellow box), favorable allele of *lcyE* (purple box), favorable allele of *crtRB1* (pink box); group 1–10 represents the chromosome 1–10.

Four BC_2_F_2_ populations with a size of 96–120 individuals were raised. Foreground selection with *InDel118* identified three genotypic classes in 1:2:1 without any segregation distortion in any of the populations (Supplementary Figure 1; Table 1). Individuals homozygous for favorable allele of *vte4* (25, 28, 29 and 32) were identified in populations of HKI161-VA, HKI163-VA, HKI193-1-VA, and HKI193-2-VA, respectively (Table 1). All the foreground positive plants for *InDel118* also possessed favorable allele of *o2, crtRB1*, and *lcyE* (Supplementary Figures 2–4; Table 1). Recurrent parent genome in phenotypically selected BC_2_F_2_ individuals ranged from 84.45 to 95.45% across the populations (Figure 1; Table 2). Based on high RPG and phenotype in relation to plant, ear and grain characteristics, three to four segregants in each of the crosses were selected. HKI161-VA-19-4-60 (95.45%) and HKI161-VA-57-5-4 (91.70%) of HKI161-VA families and HKI163-VA-8-52-26 (91.15%) and HKI163-VA-8-82-18 (90.20%) of HKI163-VA families were the best with high RPG. Among HKI193-1-VA- and HKI193-2-VA-based families, HKI193-1-VA-20-37-64 (93.35%), HKI193-1-VA-10-5-19 (91.45%), HKI193-2-VA-1-94-84 (88.75%), and HKI193-2-VA-1-94-70 (88.75%) were the most promising with higher RPG.

### Nutritional Quality Attributes of Introgressed Inbreds

ANOVA showed a significant variation among the recurrent and introgressed inbred lines for most of the quality traits except tryptophan (Supplementary Table 6). Introgressed progenies recorded higher α-tocopherol than their recurrent parents. The recurrent parents, *viz*. HKI161-VA (9.01 μg/g), HKI163-VA (7.91 μg/g), HKI193-1-VA (8.32 μg/g), and HKI193-2-VA (6.26 μg/g), recorded lower α-tocopherol. HP465-41 with higher α-tocopherol (18.38 μg/g) was used as donor parent. α-Tocopherol across introgressed progeny was significantly high (16.13 μg/g) as compared to 7.90 μg/g in the original lines. However, α-tocopherol varied from 17.51 to 19.73 μg/g (mean: 18.21 μg/g), 13.05 to 15.69 μg/g (mean: 14.4 μg/g), 16.19 to 18.14 μg/g (mean: 17.03 μg/g), and 12.87 to 15.35 μg/g (mean: 13.91 μg/g) among introgressed progenies of HKI161-VA, HKI163-VA, HKI193-1-VA, and HKI193-2-VA. HKI161-VA-57-5-4 was the best progeny with 19.73 μg/g of α-tocopherol, while other promising progenies were HKI163-VA-8-52-26 (15.69 μg/g), HKI193-1-VA-20-37-64 (18.14 μg/g), and HKI193-2-VA-1-94-84 (15.35 μg/g) (Table 3). The mean γ-tocopherol in HKI161-VA-derived progenies was 16.56 μg/g, while it was 2.59, 39.67, and 29.97 μg/g among progenies of HKI163-VA, HKI193-1-VA, and HKI193-2-VA, respectively (Table 3). The γ-tocopherol values among the original parents ranged from 7.37 to 40.62 μg/g. The mean δ-tocopherol and total tocopherol were 4.52 and 43.70 μg/g, respectively, across the introgressed progenies. These nutrients were low among the parents (4.00 and 32.60 μg/g, respectively). The concentration of β-tocopherol was quite low and could not be detected. A significant change in the proportion of different tocopherol components and total tocopherol was also observed in the improved lines. The mean α-tocopherol to total tocopherol increased from 30% among parental lines to 45% across introgressed progenies. Similarly, α-tocopherol to γ-tocopherol also recorded an enhancement to a level of 185% among the improved introgressed lines, while it was 60% among the parental lines. Zunjare et al. (2018a) reported high proA (10–12 μg/g) in the *crtRB1* and *lcyE* introgressed maize genotypes compared to 1–2 μg/g in the traditional maize. Mean proA among introgressed progenies was high (10.42 μg/g) with a range of 7.43–12.42 μg/g. Similar levels of proA were observed in the parental lines, viz. HKI161-VA (10.44 μg/g), HKI163-VA (8.37 μg/g), HKI193-1-VA (10.78 μg/g), and HKI193-2-VA (12.42 μg/g) (Table 3). Quality protein maize maize cultivars possess higher tryptophan (>0.06%) and lysine (>0.30%) contents than normal maize (tryptophan: 0.03–0.04%, lysine: 0.15–0.20%) (Hossain et al., 2018). The lysine among the MAS-derived progenies was also high with a range from 0.309 to 0.408% with a mean of 0.352%. Mean tryptophan among the progenies was high (0.083%) with a range from 0.076 to 0.092%. Similarly, high level of lysine (0.364%) and tryptophan (0.086%) was recorded among the parents (Table 3).

**Table 3:** Mean performance of *vte4* introgressed progenies and their recurrent parents for different quality parameters.

**Genotype**	**AT (μg/g)**	**Fold change**	**GT (μg/g)**	**DT (μg/g)**	**TT (μg/g)**	**AT/GT**	**AT/TT**	**proA (μg/g)**	**Lys (%)**	**Trp (%)**
HKI161-VA (RP)	9.01	–	12.72	2.18	23.91	0.71	0.38	10.44	0.331	0.091
HKI161-VA-19-4-18	17.67	1.96	18.00	3.89	39.56	0.98	0.45	10.26	0.341	0.077
HKI161-VA-19-4-54	17.51	1.94	18.12	2.70	38.33	0.97	0.46	11.30	0.376	0.079
HKI161-VA-19-4-60	17.93	1.99	16.67	1.95	36.55	1.08	0.49	10.88	0.377	0.082
HKI161-VA-57-5-4	19.73	2.19	13.46	2.20	35.39	1.47	0.56	9.68	0.394	0.092
Mean (MAS-derived progenies)	18.21	2.02	16.56	2.69	37.46	1.12	0.49	10.53	0.372	0.083
HKI163-VA (RP)	7.91	–	7.37	2.50	17.79	1.08	0.44	8.37	0.392	0.076
HKI163-VA-8-52-26	15.69	1.98	2.07	1.43	19.19	7.99	0.82	8.10	0.408	0.085
HKI163-VA-8-82-18	13.05	1.65	2.28	1.66	17.00	5.79	0.77	7.43	0.354	0.080
HKI163-VA-38-5-41	14.47	1.83	3.43	0.95	18.85	4.38	0.77	9.74	0.334	0.082
Mean (MAS-derived progenies)	14.40	1.82	2.59	1.35	18.34	6.05	0.78	8.43	0.366	0.082
HKI193-1-VA (RP)	8.32	–	40.62	6.81	55.74	0.20	0.15	10.78	0.377	0.091
HKI193-1-VA-10-5-19	16.19	1.95	48.52	6.89	71.60	0.33	0.23	11.63	0.329	0.087
HKI193-1-VA-10-5-36	17.01	2.05	40.07	7.54	64.61	0.42	0.26	11.12	0.401	0.084
HKI193-1-VA-20-37-30	16.77	2.02	33.49	7.68	57.94	0.50	0.29	11.47	0.309	0.082
HKI193-1-VA-20-37-64	18.14	2.18	36.60	6.52	61.26	0.50	0.30	10.16	0.313	0.079
Mean (MAS-derived progenies)	17.03	2.05	39.67	7.16	63.85	0.44	0.27	11.09	0.338	0.083
HKI193-2-VA (RP)	6.26	–	22.31	4.50	33.07	0.28	0.19	12.42	0.355	0.084
HKI193-2-VA-1-93-45	13.50	2.16	25.85	7.71	47.05	0.52	0.29	11.30	0.323	0.084
HKI193-2-VA-1-94-70	12.87	2.06	35.39	7.19	55.44	0.36	0.23	12.25	0.344	0.085
HKI193-2-VA-1-94-84	15.35	2.45	28.69	4.95	49.00	0.54	0.31	10.62	0.318	0.082
Mean (MAS-derived progenies)	13.91	2.22	29.97	6.61	50.50	0.47	0.28	11.39	0.328	0.084
SE_d_	1.20	–	1.60	0.99	2.63	0.67	0.02	0.51	0.014	0.006

*AT, α-tocopherol; GT, γ-tocopherol; DT, δ-tocopherol; TT, total tocopherol; proA, provitamin-A; Lys, lysine; Trp, tryptophan*.

* *HP465-41 (Donor) recorded 18.38 μg of α-tocopherol and 28.14 μg of γ-tocopherol*.

### Phenotypic Characterization of the Introgressed Inbreds

Significant variation was observed among the recurrent and introgressed inbred lines for each of the phenotypic traits (Supplementary Table 7). However, the introgressed inbred lines were comparable to their original parents for the majority of agronomic and yield characteristics (Supplementary Table 8). Plant and ear height, flowering time, and yield-related traits were predominantly *at par* with the original inbreds. Improved lines were also similar to original parents for most of the DUS traits except for (i) anthocyanin coloration of brace root in progenies of HKI161-VA and (ii) anthocyanin coloration of glume of tassel excluding base in some of the progenies of HKI161-VA, HKI163-VA, HKI193-1-VA, and HKI193-2-VA. Attitude of the leaf blade was straight in HKI163-VA, while one of the progenies showed drooping type. Similarly, anthocyanin coloration in the leaf sheath was absent in HKI163-VA, while it was present in all the progenies of the line (Supplementary Table 9).

### Nutritional Quality Attributes of Reconstituted Hybrids

Significant variation was observed among the original and reconstituted hybrids for each of the quality traits (Supplementary Table 10). However, *vte4-*based reconstituted hybrids recorded higher mean α-tocopherol (16.83 μg/g) with a range of 14.77–19.31 μg/g. The original hybrids recorded low α-tocopherol (6.72–8.67 μg/g), while it was 8.74 μg/g in the check. HQPM1-VA+VE-III recorded the highest α-tocopherol (19.31 μg/g), while the other promising hybrids were HQPM4-VA+VE-III (17.11 μg/g), HQPM5-VA+VE-I (17.60 μg/g), and HQPM7-VA+VE-II (18.03 μg/g) (Table 4; Supplementary Figure 5). Overall, there was 2.11-fold increase in α-tocopherol. The mean γ-tocopherol and total tocopherol among the reconstituted hybrids were 22.35 μg/g (range: 13.73–32.91 μg/g) and 45.20μg/g (range: 37.66–53.72 μg/g) compared to 18.78 and 31.24 μg/g in the original hybrids. The same in the check hybrids were 19.88 and 32.73 μg/g, respectively. The value of δ-tocopherol in the reconstituted hybrids was higher (7.27 μg/g) than that of the original hybrids (5.43 μg/g) and check (4.20 μg/g) (Table 4). α-, γ-, and δ-tocopherol to the total tocopherols in the original hybrids were 25, 58, and 17%, whereas an increased proportion of α-tocopherol (36%) was recorded in improved hybrids with 48 and 16% of γ- and δ-tocopherol, respectively. The ratio of α- to γ-tocopherol and α- to total tocopherol increased to 0.82 and 0.38 from 0.46 and 0.26, respectively. Improved hybrids had high proA (mean: 11.48 μg/g, range: 9.61–13.38 μg/g) similar to original hybrids (mean: 11.43 μg/g, range: 9.62–12.38 μg/g) and check (10.84 μg/g) (Table 4; Supplementary Figure 6). The lysine (mean: 0.367%, range: 0.324–0.407%) and tryptophan (mean: 0.084%, range: 0.077–0.096%) in the reconstituted hybrids were also high and *at par* with the original hybrids (lysine, mean: 0.368%, range: 0.327–0.391%; tryptophan, mean: 0.087%, range: 0.078–0.091%) and check (lysine: 0.340%, tryptophan: 0.080%) (Table 4; Supplementary Figures 7, 8). PVQ9I recorded high proA (10.84 μg/g), lysine (0.340%), and tryptophan (0.080%).

**Table 4:** Mean performance of *vte4*-derived reconstituted hybrids and their original hybrids for different quality parameters.

**Genotype**	**AT (μg/g)**	**Fold change**	**GT (μg/g)**	**DT (μg/g)**	**TT (μg/g)**	**AT/GT**	**AT/TT**	**proA (μg/g)**	**Lys (%)**	**Trp (%)**
HQPM1-VA	8.33	–	18.63	3.27	29.65	0.45	0.28	11.49	0.376	0.078
HQPM1-VA+VE-I	16.12	1.94	20.76	6.10	41.99	0.79	0.39	12.54	0.387	0.089
HQPM1-VA+VE-II	16.84	2.02	19.44	4.46	40.03	0.88	0.42	11.95	0.368	0.079
HQPM1-VA+VE-III	19.31	2.32	19.64	4.54	42.83	0.99	0.45	10.70	0.355	0.081
Mean	17.42	2.09	19.95	5.03	41.62	0.89	0.42	11.73	0.370	0.083
HQPM4-VA	8.51	–	17.42	6.87	31.41	0.49	0.27	12.38	0.327	0.091
HQPM4-VA+VE-I	15.25	1.79	24.01	8.15	46.32	0.64	0.33	12.74	0.343	0.083
HQPM4-VA+VE-II	14.77	1.74	24.13	10.80	47.71	0.62	0.31	12.22	0.380	0.086
HQPM4-VA+VE-III	17.11	2.01	22.91	8.96	47.30	0.75	0.36	10.62	0.385	0.079
Mean	15.71	1.85	23.68	9.30	47.11	0.67	0.33	11.86	0.369	0.083
HQPM5-VA	8.67	–	14.45	7.38	28.97	0.61	0.30	9.62	0.379	0.090
HQPM5-VA+VE-I	17.60	2.03	13.73	9.52	38.95	1.28	0.46	9.61	0.381	0.076
HQPM5-VA+VE-II	17.43	1.96	17.45	13.14	45.54	1.02	0.39	10.57	0.384	0.085
HQPM5-VA+VE-III	17.38	2.01	14.15	7.30	37.66	1.24	0.46	10.04	0.332	0.096
Mean	17.47	2.02	15.11	9.99	40.72	1.18	0.44	10.07	0.366	0.086
HQPM7-VA	6.72	–	24.61	4.21	34.93	0.27	0.19	12.24	0.391	0.088
HQPM7-VA+VE-I	16.78	2.50	32.91	5.05	53.72	0.52	0.31	13.38	0.324	0.087
HQPM7-VA+VE-II	18.03	2.72	30.50	5.30	53.22	0.60	0.34	13.06	0.407	0.094
HQPM7-VA+VE-III	15.31	2.28	28.53	3.93	47.15	0.54	0.32	10.31	0.362	0.077
Mean	16.71	2.49	30.65	4.76	51.36	0.55	0.32	12.25	0.364	0.086
PVQ9I (Commercial check)	8.74	–	19.88	4.20	32.73	0.44	0.27	10.84	0.340	0.080
SE_d_	0.93		1.16	0.63	2.22	0.06	0.02	0.24	0.020	0.003

*Lys, lysine; Trp, tryptophan; proA, provitamin-A; AT, α-tocopherol; GT, γ-tocopherol; DT, δ-tocopherol; TT, total tocopherol*.

### Grain Yield and Related Traits of Reconstituted Hybrids

Significant variation was observed among the original and reconstituted hybrids for each of the phenotypic traits (Supplementary Table 11). However, original and reconstituted hybrids for different phenotypic traits, the grain yield and yield-attributing traits of most of the reconstituted hybrids were *at par* or higher with their respective original hybrids (Supplementary Table 12). The improved multinutrient-rich hybrids in the genetic background of HQPM1-VA, HQPM4-VA, HQPM5-VA, and HQPM7-VA recorded the mean grain yield of 8,530, 7,365, 8,332, and 7,896 kg/ha, while the same in the original hybrids were 8,207 kg/ha, 7,084 kg/ha, 8,546 kg/ha, and 7,548 kg/ha, respectively. Further, all the reconstituted hybrids recorded a higher yield than the commercial check, PVQ9I (6,337 kg/ha).

### Distinctness, Uniformity, and Stability Traits of Reconstituted Hybrids

Each of the reconstituted hybrids was characterized for DUS traits. Original and improved hybrids were similar for most of the DUS traits except (i) HQPM4-VA+VE-I for anthocyanin coloration of glume excluding base and color of top of grains, (ii) HQPM5-VA+VE for anthocyanin coloration of brace root and color of grain, (iii) HQPM7-VA+VE-I, -II, and -III versions for anthocyanin coloration of brace root, (iv) HQPM7-VA+VE-III for color of top of grains and (v) HQPM5-VA+VE-II, HQPM5-VA+VE-III, HQPM7-VA+VE-I, and HQPM7-VA+VE-II for the width of leaf blade (Supplementary Table 13).

## Discussion

Traditional maize hybrids are deficient in essential nutrients including balanced protein enriched with lysine and tryptophan, and vitamins such as vitamin-A and vitamin-E (Prasanna et al., 2020). Maize mutants rich in these nutrients are available, *viz*. (i) *o2* mutant with higher lysine and tryptophan (Mertz et al., 1964; Vasal, 2000), (ii) *crtRB1* and *lcyE* mutants for high proA (Harjes et al., 2008; Yan et al., 2010), and (iii) *vte4* mutant for higher vitamin-E or α-tocopherol (Li et al., 2012). The development of multinutrient maize cultivars with enriched lysine, tryptophan, vitamin-A, and vitamin-E holds immense significance to human health as multiple types of malnutrition can be addressed in a more sustainable way (Bouis, 2018). Stacking of genes in a single genetic background is challenging as it takes a long time and requires costly phenotyping in a large scale. However, MABB approach can achieve the goal with reduced time and cost (Collard et al., 2005; Muthusamy et al., 2014; Zunjare et al., 2018b). Hence, the study was undertaken to combine the mutant alleles of *vte4, o2, crtRB1*, and *lcyE* into one background to develop multinutrient-rich maize hybrids using the molecular breeding method. Four popular QPM hybrids already improved for proA (Zunjare et al., 2018a) were targeted for α-tocopherol enrichment.

### Segregation of Target Genes

Donor and recurrents parent carried the favorable allele of *vte4* and *o2*, respectively, and both shared the favorable alleles of *crtRB1* and *lcyE*. Gene-based markers, *viz*. *InDel118, phi057, 3*′*TE-InDel*, and *5*′*TE-InDel*, were used for foreground selection of favorable alleles of *vte4, o2, crtRB1*, and *lcyE*, respectively. All of these markers are located within the target genes. Gene-based markers have a significant advantage over linked markers, which may lead to the selection of false-positive individuals due to genetic recombination between the target gene and the linked marker (Collard et al., 2005). The use of these gene-based markers ensures the accurate transfer of the target gene. Several researchers have successfully used these markers, *viz*. *vte4* (Feng et al., 2015), *o2* (Hossain et al., 2018), *crtRB1* (Muthusamy et al., 2014; Zunjare et al., 2017, 2018a), and *lcyE* (Zunjare et al., 2018a,c) in MABB programme. *vte4* gene followed the Mendelian segregation in BC_1_F_1_, BC_2_F_1_, and BC_2_F_2_ generations. *o2* showed expected segregation in three of the BC_1_F_1_ populations, whereas one BC_1_F_1_ population (HKI193-2 based) showed a strong segregation distortion (SD). Hossain et al. (2018) also reported SD in some of the backcross populations segregating for *o2*. Distortion of a locus could be attributed to the existence of gametophytic factors, male sterility, mutants like defective kernel and embryo-specific mutation (Neuffer et al., 1997; Lu et al., 2002). The SD is possibly attributed to the presence of such type of loci in the genetic background of HKI193-2, which was quite different from the other three genetic backgrounds. Though in our populations, *crtRB1* and *lcyE* did not segregate, strong SD for these two genes were reported in previous studies (Babu et al., 2013; Muthusamy et al., 2014; Liu et al., 2015; Zunjare et al., 2017, 2018a).

### Recovery of Background Genome

Recovery of background genome during conventional backcross breeding approach occurs at the rate of 1 – (1/2)^n+1^, where “*n*” denotes the number of backcrossing (Allard, 1999). Here, marker-assisted background selection along with phenotypic selection helped in a rapid recovery of background genome in selected progenies. Recovery of RPG of selected progenies was 84.45–90.0% with the highest being 95.45%. Zunjare et al. (2018a) could also recover 83.86–92.98% of the background genome by using 114–133 polymorphic markers in maize. Higher RPG could be recovered in just two backcrosses compared to conventional backcross breeding programme (Muthusamy et al., 2014; Hossain et al., 2018; Sarika et al., 2018; Zunjare et al., 2018a).

### Nutritional Enhancement in MAS-Derived Genotypes

Though all forms (α-, β-, γ-, and δ-) of tocopherols possess vitamin-E activity, α-tocopherol fractions possess six times more vitamin-E activity than γ-tocopherol (Das et al., 2020). Human liver preferentially absorbs α-tocopherol due to efficient hepatic α-tocopherol transfer protein (HTP) resulting in 10 times more accumulation than γ-tocopherol (Frank et al., 2012). Increased level of α-tocopherol with an increased proportion of α-/γ-tocopherol and α-/total tocopherol was recorded in each of the introgressed families. Maize genotypes carrying favorable haplotype of *vte4* was reported to have a higher proportion of α-/γ-tocopherol and α-/total tocopherol than genotypes with unfavorable allele (Das et al., 2019a). Kernel α-tocopherol was enhanced by 1.82- to 2.22-fold in the MAS-derived inbreds and 1.85- to 2.49-folds among the reconstituted hybrids. Feng et al. (2015) reported 0.95- to 2.64-fold increase in α-tocopherol in the *vte4* introgressed sweet corn inbreds, whereas Das et al. (2018) and Li et al. (2012) reported much higher concentration of α-tocopherol (4.3- and 3.2-fold, respectively) in selected maize genotypes with a favorable allele of *vte4*. *InDel118* affects the level of transcription, and *InDel7* regulates the translational efficiency of the *vte4*, thereby ensuring more kernel α-tocopherol (Li et al., 2012). In general, γ-tocopherol, δ-tocopherol, and total tocopherol were also increased in the *vte4*-based genotypes. This could be due to the higher flux of initial precursors of the pathway due to more conversion of γ-tocopherol to α-tocopherol in the last step of the tocopherol pathway. It is important to mention here that γ-tocopherol and δ-tocopherol also possess important functions as antioxidants (Evans et al., 2002). Thus, simultaneous enhancement of γ-tocopherol, δ-tocopherol, and total tocopherol along with α-tocopherol is beneficial for human health (Das et al., 2020). Kernel lysine and tryptophan in the introgressed progenies as well as in reconstituted hybrids were comparable to their respective recipient parents. Several mechanisms contribute to the enhancement of lysine and tryptophan in *o2* genotypes, *viz*. (i) lysine-deficient zein fraction of protein is reduced with a consequent increase in lysine-rich non-zein proteins (Habben et al., 1994); (ii) reduction of transcription of lysine keto-reductase, which is a lysine-catabolizing enzyme (Kemper et al., 1999); and (iii) greater accumulation of several lysine-rich enzymes and proteins (Jia et al., 2013). The presence of favorable allele of *crtRB1* and *lcyE* also led to the comparable but high concentration of proA in the introgressed progenies and hybrids. *lcyE* and *crtRB1*enhance kernel proA by regulating the pathway branching and hydroxylation, respectively (Harjes et al., 2008; Yan et al., 2010). Mutant *crtRB1* and *lcyE* alleles produce lesser amount β-hydroxylase and β-cyclase enzymes, respectively, than the wild type, and result in a reduced conversion of β-carotene to downstream components and shifting more lycopene flux from α-branch to β-branch of the pathway (Harjes et al., 2008; Vallabhaneni et al., 2009; Yan et al., 2010). Though the frequency of co-occurrence of favorable alleles of *crtRB1* and *lcyE* is very low in the maize germplasm (Azmach et al., 2013; Babu et al., 2013; Muthusamy et al., 2014; Gebremeskel et al., 2018), the greater combined effect of both over individual allele has been reported in a number of studies (Azmach et al., 2013; Zunjare et al., 2017; Gebremeskel et al., 2018). Further, the analysis revealed that a greater proportion of variations in nutritional quality traits were genetic in nature, and a minor variation was due to G × E interaction (α-T: 3.14%, γ-T: 3.40%, δ-T: 4.13%, TT: 3.32%, proA: 12.98%, lysine: 22.89%, and tryptophan: 10.77%). This suggested that these nutritional traits would not vary drastically when grown in a different environment (Hossain et al., 2018; Zunjare et al., 2018a). Recommended dietary allowance (RDA) for vitamin-E and vitamin-A is 10 mg/day and 4,800 μg/day for adults in humans, respectively (ICMR, 2010). While the RDA for lysine is 2,100 mg/day, the same for tryptophan is 288 mg/day for an adult of 60 kg weight (Gupta et al., 2015). Thus, the consumption of these biofortified maize hybrids (200 gm/day) can meet approximately the 32, 50, 38, and 55% of RDA for vitamin-E, vitamin-A, lysine, and tryptophan, respectively, as compared to 16, 8, 20, and 28% of normal maize (Dube et al., 2018).

### Background Effect and Gene Interaction

Increased α-tocopherol content in each of these introgressed families provides direct evidence of the major effect of *vte4* on the accumulation of α-tocopherol. However, a variation for kernel α-tocopherol within families even in the presence of the same favorable allele of *vte4* could be due to background effect and differential interaction with genes in the pathway (Babu et al., 2013; Muthusamy et al., 2014). In contrast to the report of Li et al. (2012), introgressed progenies also revealed an increased quantity of γ-tocopherol and total tocopherol. Similar results were also reported by Feng et al. (2015) in the MAS-derived sweet corn inbreds with *vte4*. Most of the introgressed progenies except HKI161-VA-57-5-4 recorded kernel α-tocopherol concentration less than donor parent, which suggested the involvement of other minor quantitative trait loci (QTL)/genes, which possibly were not transferred from the donor line due to stringent background selection (Wong et al., 2003; Chander et al., 2008; Zhou et al., 2012). Rocheford et al. (2002), Wong et al. (2003), and Feng et al. (2013) detected minor QTL, which affects tocopherol composition in maize kernel. Though the selection for yield as such was not applied during the introgression process, the resemblance of reconstituted hybrids with original hybrids for grain yield was due to the high recovery of loci responsible for yield in recurrent parent through background selection (Hossain et al., 2018). However, interactions of a small fraction of the introgressed donor genome with the RPG resulted in a minor but significant difference in the grain yield of improved hybrids and respective original hybrids (Choudhary et al., 2014). Moreover, as grain yield is a quantitative trait, these variations could also be the influence of environment and genotype × environment. The α-tocopherol content of most of the reconstituted hybrids deviated from the mid-parent value of the introgressed inbred lines, suggesting interactions of various loci affecting tocopherol accumulation and non-additive gene action in the hybrids. Non-additive gene action for various tocopherol components in maize kernel has been reported by Das et al. (2019b). These newly derived hybrids rich in vitamin-E, vitamin-A, lysine, and tryptophan were high-yielding, and superior to the commercial heck, PVQ9I. These multinutrient-rich hybrids assume great significance in alleviating malnutrition through a holistic approach.

## Conclusions

The benefit of biofortified maize hybrids for human health has been well-documented in several countries. However, studies on biofortification for kernel vitamin-E are limited in the case of maize. So far, maize hybrids rich in proA, lysine, and tryptophan have been developed and commercialized. However, this study is the first in reporting vitamin-E enrichment along with proA, lysine, and tryptophan in maize. Here, we demonstrated for the first time the development of multinutrient-rich maize with high vitamin-E, vitamin-A, lysine, and tryptophan by stacking of favorable alleles of *vte4, o2, crtRB1*, and *lcyE* in maize inbreds using MABB. These newly developed high-yielding maize hybrids with higher vitamin-E, vitamin-A, lysine, and tryptophan will help in addressing malnutrition in a more holistic and sustainable way.

## Data Availability Statement

The original contributions presented in the study are included in the article/Supplementary Material, further inquiries can be directed to the corresponding author/s.

## Author Contributions

Foreground and background selection in backcross populations was done by AD and HC. Phenotyping of introgressed inbreds and reconstituted hybrids was done by MG. Generation of backcross populations was done by VM and AD. Data analysis and interpretation were made by RZ. Biochemical analysis of tocopherols was performed by AD and HC. Biochemical analysis of carotenoids and amino acids was done by AB. Data recording of field experiments was done by VB and GC. Evaluation of multilocation trials was done by JB and SG. Protocol standardization was made by SS. Drafting of manuscript was made by AD, MG, VM, and FH. Design of experiments was made by FH, VM, and HG. Generation of funds was done by FH and HG. All authors contributed to the article and approved the submitted version.

## Conflict of Interest

The authors declare that the research was conducted in the absence of any commercial or financial relationships that could be construed as a potential conflict of interest.
