# Supplementary material for: Development of Maize Hybrids With Enhanced Vitamin-E, Vitamin-A, Lysine, and Tryptophan Through Molecular Breeding
Source: Front Plant Sci. 2021 Jul 21;12:659381. doi: 10.3389/fpls.2021.659381 (PMC8335160; doi:10.3389/fpls.2021.659381)
Supplement: Supplementary file 1 [file Data_Sheet_1.docx]

**Table S1: Details of the recurrent and donor parents used in the study**

| **S.**  **No.** | **Hybrid** | **Parents** | **Maturity** | **Year** | **Zone** | **Improved version of pro A** | **Improved Parents** |
| --- | --- | --- | --- | --- | --- | --- | --- |
| 1 | HQPM1 | HKI-193-1 × HKI-163 | Late | 2005 | Across the country | HQPM1-VA | HKI-193-1-VA × HKI-163-VA |
| 2 | HQPM4 | HKI-193-2 × HKI-161 | Late | 2010 | Across the country | HQPM4-VA | HKI-193-2-VA × HKI-161-VA |
| 3 | HQPM5 | HKI-163 × HKI-161 | Late | 2007 | Across the country | HQPM5-VA | HKI-163-VA × HKI-161-VA |
| 4 | HQPM7 | HK1-193-1 × HKI-161 | Late | 2008 | Peninsular Zone | HQPM7-VA | HK1-193-1-VA × HKI-161-VA |

**Table S2: Pedigree of the recurrent and donor parents used in the study**

| **No.** | **Name** | **Derived from** | **Source institute** |
| --- | --- | --- | --- |
| Recurrent parents | | | |
| 1. | HKI161-VA | (HKI161/// HP704-23)-**⊗**-**⊗**-**⊗**-**⊗-**­**⊗-**­**⊗-⊗-⊗** | IARI, New Delhi |
| 2. | HKI163-VA | (HKI163///HP704-22)-**⊗**-**⊗**-**⊗**-**⊗-**­**⊗-**­**⊗-⊗-⊗** | IARI, New Delhi |
| 3. | HKI193-1-VA | (HKI193-1///HP704-23)-**⊗**-**⊗**-**⊗**-**⊗-**­**⊗-**­**⊗-⊗-⊗** | IARI, New Delhi |
| 4. | HKI193-2-VA | (HKI193-2///HP704-22)-**⊗**-**⊗**-**⊗**-**⊗-**­**⊗-**­**⊗-⊗-⊗** | IARI, New Delhi |
| Donor parent | | | |
| 1. | HP465-41 | KUI carotenoid syn-FS25-3-2-B-B-B/(KU1409/DE3/KU1409)S2-18-2-B | CIMMYT, Mexico |

**Table S3: List of markers used for foreground selection**

| **S. No.** | **Gene** | **Bin location** | **Marker** | **Primer sequence** | **Primer** |
| --- | --- | --- | --- | --- | --- |
| 1 | *VTE4* | 5.06 | *InDel118* | AAAGCACTTACATCATGGGAAAC | Forward |
|  |  |  |  | TTGGTGTAGCTCCGATTTGG | Reverse |
|  |  |  | *InDel7* | GCCGGCACCTCTACTTTAT | Forward |
|  |  |  |  | AGGACTGGGAGCAATGGAG | Reverse |
| 2 | *crtRB1* | 10.05 | *3′TE-InDel* | ACACCACATGGACAAGTTCG | Forward |
|  |  |  |  | ACACTCTGGCCCATGAACAC | Reverse1 |
|  |  |  |  | ACAGCAATACAGGGGACCAG | Reverse2 |
| 3 | *lcyE* | 8.05 | *5′TE-InDel* | AAGCAGGGAAGACATTCCAG | Forward |
|  |  |  |  | GAGAGGGAGACGACGAGACAC | Reverse |
| 4 | *Opaque2* | 7.01 | *phi057* (SSR) | CTCATCAGTGCCGTCGTCCAT | Forward |
|  |  |  |  | CAGTCGCAAGAAACCGTTGCC | Reverse |

**Table S4: Percent polymorphism and distribution of SSRs used for background selection**

| **Linkage group (LG)** | **No. of primers**  **Screened** | **No. of polymorphic markers** | | | |
| --- | --- | --- | --- | --- | --- |
|  |  | **HKI161-PV ×**  **HP465-41** | **HKI163-PV ×**  **HP465-41** | **HKI193-1-PV ×**  **HP465-41** | **HKI193-2-PV ×**  **HP465-41** |
| LG1 | 38 | 8 | 9 | 13 | 10 |
| LG2 | 28 | 4 | 10 | 10 | 13 |
| LG3 | 21 | 15 | 8 | 9 | 7 |
| LG4 | 25 | 15 | 10 | 5 | 10 |
| LG5 | 22 | 12 | 9 | 10 | 5 |
| LG6 | 34 | 7 | 5 | 11 | 9 |
| LG7 | 32 | 12 | 8 | 8 | 8 |
| LG8 | 25 | 8 | 8 | 4 | 5 |
| LG9 | 28 | 8 | 9 | 15 | 9 |
| LG10 | 32 | 14 | 5 | 4 | 5 |
| **Total** | **285** | **103**  **(36.14%)** | **81**  **(28.42%)** | **89**  **(31.22%)** | **81**  **(28.42%)** |

**Table S5: Details of the reconstituted hybrids and the checks evaluated in the study**

| **S. No.** | **Hybrid** | **Parentage** |
| --- | --- | --- |
| 1 | HQPM1-VA | HKI193-1-VA × HKI163-VA |
| 2 | HQPM1-VA+VE-I | HKI193-1-VA-20-37-64 × HKI163-VA-38-5-41 |
| 3 | HQPM1-VA+VE-II | HKI163-VA-38-5-41 × HKI193-1-VA-10-5-36 |
| 4 | HQPM1-VA+VE-III | HKI193-1-VA-20-37-64 × HKI163-VA-8-52-26 |
| 5 | HQPM4-VA | HKI193-2-PVA × HKI161-PVA |
| 6 | HQPM4-VA+VE-I | HKI193-2-VA-1-94-84 × HKI161-VA-57-5-4 |
| 7 | HQPM4-VA+VE-II | HKI193-2-VA-1-94-70 × HKI161-VA-57-5-4 |
| 8 | HQPM4-VA+VE-III | HKI193-2-VA-1-93-45 × HKI161-VA-57-5-4 |
| 9 | HQPM5-VA | HKI163-PVA × HKI161-PVA |
| 10 | HQPM5-VA+VE-I | HKI161-VA-19-4-18 × HKI163-PVA+PVE-II |
| 11 | HQPM5-VA+VE-II | HKI161-VA-19-4-54 × HKI163-PVA+PVE-III |
| 12 | HQPM5-VA+VE-III | HKI161-VA-19-4-60 × HKI163-PVA+PVE-I |
| 13 | HQPM7-VA | HKI193-1-PVA × HKI161-PVA |
| 14 | HQPM7-VA+VE-I | HKI161-VA-19-4-18 × HKI193-1-VA-20-37-30 |
| 15 | HQPM7-VA+VE-II | HKI161-VA-19-4-60 × HKI193-1-VA-10-5-19 |
| 16 | HQPM7-VA+VE-III | HKI161-VA-19-4-54 × HKI193-1-VA-10-5-36 |
| 17 | PVQ9I | VQL1-PV × VQL2-PV |

QPM: quality protein maize; proA: provitamin-A; proE: provitamin-E

**Table S6: ANOVA for different quality parameters among introgressed and original inbreds**

| **Source of variation** | **df** | **Mean sum of square** | | | | | | | | |
| --- | --- | --- | --- | --- | --- | --- | --- | --- | --- | --- |
|  |  | **AT** | **GT** | **DT** | **TT** | **AT/GT** | **AT/TT** | **ProA** | **Lysine** | **Tryptophan** |
| Replicates | 1 | 0.22 | 1.01 | 4.69* | 5.46 | 0.01 | 0.00004 | 0.13 | 0.0002 | 0.00002 |
| Treatment | 17 | 32.05** | 413.99** | 10.07** | 613.36** | 9.55** | 0.08** | 3.76** | 0.0021** | 0.00004 |
| Error | 17 | 1.44 | 2.56 | 0.99 | 6.92 | 0.45 | 0.00033 | 0.26 | 0.0002 | 0.00003 |
| Total | 35 | 16.27 | 202.36 | 5.51 | 301.43 | 4.85 | 0.04 | 1.95 | 0.0011 | 0.00004 |

* & **: Significance at 5% and 1% level of significance, respectively, df: degree of freedom, AT: α-tocopherol; GT: γ-tocopherol; DT: δ-tocopherol; TT: total tocopherol; ProA: provitamin-A,

**Table S7: ANOVA for different yield and morphological traits among introgressed and original inbreds**

| **Source of variation** | **df** | **Mean sum of square** | | | | | | | | | |
| --- | --- | --- | --- | --- | --- | --- | --- | --- | --- | --- | --- |
|  |  | **GY** | **MF** | **FF** | **PH** | **EH** | **EL** | **EG** | **NR** | **NKR** | **100KW** |
| Replicates | 1 | 4400.11 | 4.69* | 1.78 | 16.00 | 9.00 | 0.07 | 0.01 | 0.018 | 0.13 | 0.004 |
| Treatment | 17 | 205652.35** | 10.07** | 7.70** | 152.24** | 106.29** | 2.10** | 0.23** | 1.35** | 4.73** | 42.15** |
| Error | 17 | 12673.70 | 0.99 | 1.42 | 5.41 | 3.05882 | 0.12 | 0.07 | 0.11 | 0.28 | 0.14 |
| Total | 35 | 106169.80 | 5.51 | 4.48 | 77.03 | 53.37 | 1.08 | 0.15 | 0.71 | 2.43 | 20.55 |

* & **: Significance at 5% and 1% level of significance, respectively, df: degree of freedom, GY: grain yield; MF: days to 50% male flowering; FF: days to 50% female flowering; PH: plant height; EH: ear height; EL: ear length; EG: ear girth; NR: Number of rows; NKR: number of kernels per row; HKW: 100 kernel weight

**Table S8: Mean performance of *vte4* introgressed progenies and their recurrent parents for grain yield and flowering time**

| **Genotype** | **GY**  **(kg/ha)** | **MF**  **(days)** | **FF**  **(days)** | **PH**  **(cm)** | **EH**  **(cm)** | **EL**  **(cm)** | **EG**  **(cm)** | **NR**  **(No.)** | **NKR**  **(No.)** | **100KW**  **(gram)** |
| --- | --- | --- | --- | --- | --- | --- | --- | --- | --- | --- |
| **HKI161-VA (RP)** | 2821 | 55.5 | 59.0 | 177.0 | 74.5 | 13.0 | 2.9 | 12.0 | 20.9 | 29.7 |
| HKI161-VA-19-4-18 | 3030 | 56.0 | 59.5 | 181.5 | 75.0 | 13.1 | 3.3 | 12.6 | 21.4 | 29.1 |
| HKI161-VA-19-4-54 | 2851 | 56.0 | 58.5 | 170.0 | 70.5 | 11.8 | 3.0 | 12.2 | 19.1 | 28.4 |
| HKI161-VA-19-4-60 | 2751 | 55.5 | 58.5 | 176.0 | 75.0 | 12.5 | 3.0 | 12.4 | 19.6 | 29.0 |
| HKI161-VA-57-5-4 | 2685 | 59.5 | 62.0 | 179.5 | 76.5 | 13.6 | 3.6 | 12.8 | 21.5 | 30.2 |
| **Mean** | **2829** | **56.8** | **59.6** | **176.8** | **74.3** | **12.7** | **3.2** | **12.5** | **20.4** | **29.1** |
| **HKI163-VA (RP)** | 2675 | 63.5 | 65.5 | 183.0 | 80.0 | 13.0 | 2.8 | 12.6 | 21.6 | 28.0 |
| HKI163-VA-8-52-26 | 2460 | 60.5 | 63.0 | 187.0 | 85.0 | 13.3 | 3.0 | 13.0 | 21.8 | 28.1 |
| HKI163-VA-8-82-18 | 2671 | 60.0 | 62.5 | 181.0 | 80.0 | 12.0 | 2.6 | 12.3 | 19.6 | 26.7 |
| HKI163-VA-38-5-41 | 2610 | 58.0 | 61.5 | 174.0 | 74.5 | 11.0 | 2.6 | 12.0 | 19.0 | 27.4 |
| **Mean** | **2580** | **59.5** | **62.3** | **180.7** | **79.8** | **12.1** | **2.7** | **12.4** | **20.1** | **27.4** |
| **HKI193-1-VA (RP)** | 2115 | 59.5 | 63.0 | 162.0 | 63.5 | 11.1 | 2.5 | 11.0 | 18.4 | 20.8 |
| HKI193-1-VA-10-5-19 | 2280 | 61.5 | 64.0 | 166.5 | 66.5 | 10.8 | 2.6 | 11.1 | 18.3 | 20.0 |
| HKI193-1-VA-10-5-36 | 2335 | 57.0 | 60.5 | 162.0 | 63.0 | 11.1 | 2.4 | 10.4 | 17.4 | 19.9 |
| HKI193-1-VA-20-37-30 | 2263 | 58.5 | 61.5 | 161.5 | 59.5 | 10.8 | 2.4 | 10.1 | 17.1 | 19.3 |
| HKI193-1-VA-20-37-64 | 2048 | 60.5 | 63.0 | 154.5 | 58.5 | 10.3 | 2.4 | 11.0 | 18.4 | 21.5 |
| **Mean** | **2231** | **59.4** | **62.3** | **161.1** | **61.9** | **10.8** | **2.4** | **10.6** | **17.8** | **20.1** |
| **HKI193-2-VA (RP)** | 2185 | 57.0 | 59.5 | 171.5 | 74.5 | 11.8 | 2.7 | 11.4 | 20.4 | 18.9 |
| HKI193-2-VA-1-93-45 | 1996 | 60.5 | 62.5 | 167.5 | 70.0 | 12.0 | 2.4 | 11.9 | 21.4 | 19.4 |
| HKI193-2-VA-1-94-70 | 2195 | 59.0 | 61.5 | 174.0 | 73.0 | 13.0 | 3.0 | 12.2 | 20.6 | 19.3 |
| HKI193-2-VA-1-94-84 | 2063 | 59.5 | 60.5 | 173.5 | 76.5 | 12.9 | 2.7 | 12.1 | 21.1 | 19.0 |
| **Mean** | **2084** | **59.7** | **61.5** | **171.7** | **73.2** | **12.6** | **2.7** | **12.1** | **21.0** | **19.2** |
| **SE_d_** | 112.58 | 0.99 | 1.19 | 2.33 | 1.75 | 0.35 | 0.26 | 0.33 | 0.53 | 0.38 |

GY: grain yield; MF: days to 50% male flowering; FF: days to 50% female flowering; PH: plant height; EH: ear height; EL: ear length; EG: ear girth; NR: Number of rows; NKR: number of kernels per row; HKW: 100 kernel weight

**Table S9: DUS characteristics of *vte4* introgressed progenies and their recurrent parents**

| **Genotypes** | **1** | **2** | **3** | **4** | **5** | **6** | **7** | **8** | **9** | **10** | **11** | **12** | **13** | **14** | **15** | **16** |
| --- | --- | --- | --- | --- | --- | --- | --- | --- | --- | --- | --- | --- | --- | --- | --- | --- |
| **HKI161-VA (RP)** | Wide | Drooping | Present | Medium | Absent | Present | Present | Dense | Narrow | Straight | Medium | Present | Present | Medium | Long | Medium |
| HKI161-VA-19-4-18 | Wide | Drooping | Absent | Medium | Absent | Present | Present | Dense | Narrow | Straight | Medium | Present | Present | Medium | Long | Medium |
| HKI161-VA-19-4-54 | Wide | Drooping | Absent | Medium | Absent | Present | Present | Dense | Narrow | Straight | Medium | Present | Present | Medium | Long | Medium |
| HKI161-VA-19-4-60 | Wide | Drooping | Absent | Medium | Absent | Present | Present | Dense | Narrow | Straight | Medium | Present | Present | Medium | Long | Medium |
| HKI161-VA-57-5-4 | Wide | Drooping | Absent | Medium | Absent | Absent | Present | Dense | Narrow | Straight | Medium | Present | Present | Medium | Long | Medium |
|  |  |  |  |  |  |  |  |  |  |  |  |  |  |  |  |  |
| **HKI163-VA (RP)** | Small | Straight | Absent | Late | Absent | Absent | Absent | Dense | Narrow | Straight | Late | Present | Absent | Medium | Long | Medium |
| HKI163-VA-8-52-26 | Small | Drooping | Absent | Late | Absent | Present | Absent | Dense | Narrow | Straight | Late | Present | Present | Medium | Long | Medium |
| HKI163-VA-8-82-18 | Small | Straight | Absent | Late | Absent | Present | Absent | Dense | Narrow | Straight | Late | Present | Present | Medium | Long | Medium |
| HKI163-VA-38-5-41 | Small | Straight | Absent | Late | Absent | Absent | Absent | Dense | Narrow | Straight | Late | Present | Present | Medium | Long | Medium |
|  |  |  |  |  |  |  |  |  |  |  |  |  |  |  |  |  |
| **HKI193-1-VA (RP)** | Wide | Drooping | Absent | Late | Absent | Present | Present | Dense | Narrow | Straight | Late | Absent | Present | Medium | Long | Medium |
| HKI193-1-VA-10-5-19 | Wide | Drooping | Absent | Late | Absent | Present | Present | Dense | Narrow | Straight | Late | Absent | Present | Medium | Long | Medium |
| HKI193-1-VA-10-5-36 | Wide | Drooping | Absent | Late | Absent | Present | Present | Dense | Narrow | Straight | Late | Absent | Present | Medium | Long | Medium |
| HKI193-1-VA-20-37-30 | Wide | Drooping | Absent | Late | Absent | Present | Present | Dense | Narrow | Straight | Late | Absent | Present | Medium | Long | Medium |
| HKI193-1-VA-20-37-64 | Wide | Drooping | Absent | Late | Absent | Absent | Present | Dense | Narrow | Straight | Late | Absent | Present | Medium | Long | Medium |
|  |  |  |  |  |  |  |  |  |  |  |  |  |  |  |  |  |
| **HKI193-2-VA (RP)** | Small | Drooping | Absent | Late | Absent | Absent | Present | Dense | Wide | Straight | Late | Present | Present | Medium | Long | Medium |
| HKI193-2-VA-1-93-45 | Small | Drooping | Absent | Late | Absent | Present | Present | Dense | Wide | Straight | Late | Present | Present | Medium | Long | Medium |
| HKI193-2-VA-1-94-70 | Small | Drooping | Absent | Late | Absent | Absent | Present | Dense | Wide | Straight | Late | Present | Present | Medium | Long | Medium |
| HKI193-2-VA-1-94-84 | Small | Drooping | Absent | Late | Absent | Absent | Present | Dense | Wide | Straight | Late | Present | Present | Medium | Long | Medium |

| **Genotype** | **17** | **18** | **19** | **20** | **21** | **22** | **23** | **24** | **25** | **26** | **27** | **28** | **29** | **30** | **31** |
| --- | --- | --- | --- | --- | --- | --- | --- | --- | --- | --- | --- | --- | --- | --- | --- |
| **HKI161-VA (RP)** | Medium | Medium | Small | Conico-cylindrical | Medium | Flint | Orange | Absent | Straight | Absent | Absent | Absent | Present | Round | Large |
| HKI161-VA-19-4-18 | Medium | Medium | Small | Conico-cylindrical | Medium | Flint | Orange | Absent | Straight | Absent | Absent | Absent | Present | Round | Large |
| HKI161-VA-19-4-54 | Medium | Medium | Small | Conico-cylindrical | Medium | Flint | Orange | Absent | Straight | Absent | Absent | Absent | Present | Round | Large |
| HKI161-VA-19-4-60 | Medium | Medium | Small | Conico-cylindrical | Medium | Flint | Orange | Absent | Straight | Absent | Absent | Absent | Present | Round | Large |
| HKI161-VA-57-5-4 | Medium | Medium | Small | Conico-cylindrical | Medium | Flint | Orange | Absent | Straight | Absent | Absent | Absent | Present | Round | Large |
|  | | | | | | | | | | | | | | | |
| **HKI163-VA (RP)** | Medium | Medium | Small | Conico-cylindrical | Medium | Semi-flint | Yellow | Absent | Straight | Absent | Absent | Absent | Present | Round | Medium |
| HKI163-VA-8-52-26 | Medium | Medium | Small | Conico-cylindrical | Medium | Semi-flint | Yellow | Absent | Straight | Absent | Absent | Absent | Present | Round | Medium |
| HKI163-VA-8-82-18 | Medium | Medium | Small | Conico-cylindrical | Medium | Semi-flint | Yellow | Absent | Straight | Absent | Absent | Absent | Present | Round | Medium |
| HKI163-VA-38-5-41 | Medium | Medium | Small | Conico-cylindrical | Medium | Semi-flint | Yellow | Absent | Straight | Absent | Absent | Absent | Present | Round | Medium |
|  | | | | | | | | | | | | | | | |
| **HKI193-1-VA (RP)** | Medium | Medium | Small | Conico-cylindrical | Medium | Semi-flint | Yellow | Absent | Straight | Absent | Absent | Absent | Present | Round | Small |
| HKI193-1-VA-10-5-19 | Medium | Medium | Small | Conico-cylindrical | Medium | Semi-flint | Yellow | Absent | Straight | Absent | Absent | Absent | Present | Round | Small |
| HKI193-1-VA-10-5-36 | Medium | Medium | Small | Conico-cylindrical | Medium | Semi-flint | Yellow | Absent | Straight | Absent | Absent | Absent | Present | Round | Small |
| HKI193-1-VA-20-37-30 | Medium | Medium | Small | Conico-cylindrical | Medium | Semi-flint | Yellow | Absent | Straight | Absent | Absent | Absent | Present | Round | Small |
| HKI193-1-VA-20-37-64 | Medium | Medium | Small | Conico-cylindrical | Medium | Semi-flint | Yellow | Absent | Straight | Absent | Absent | Absent | Present | Round | Small |
|  | | | | | | | | | | | | | | | |
| **HKI193-2-VA (RP)** | Medium | Medium | Small | Conico-cylindrical | Medium | Semi-flint | Yellow cap | Absent | Straight | Absent | Absent | Absent | Present | Round | Small |
| HKI193-2-VA-1-93-45 | Medium | Medium | Small | Conico-cylindrical | Medium | Semi-flint | Yellow | Absent | Straight | Absent | Absent | Absent | Present | Round | Small |
| HKI193-2-VA-1-94-70 | Medium | Medium | Small | Conico-cylindrical | Medium | Semi-flint | Yellow | Absent | Straight | Absent | Absent | Absent | Present | Round | Small |
| HKI193-2-VA-1-94-84 | Medium | Medium | Small | Conico-cylindrical | Medium | Semi-flint | Yellow | Absent | Straight | Absent | Absent | Absent | Present | Round | Small |

1: Leaf: Angle between blade and stem (on leaf just above upper ear), 2: Leaf: Attitude of blade (on leaf just above upper ear), 3: Stem: Anthocyanin colouration of brace root, 4: Time of anthesis (on middle third of main axis 50 % of plants), 5: Tassel: Anthocyanin colouration at base of glume (in middle third of main axis), 6: Tassel: Anthocyanin colouration excluding base (in middle third of main axis), 7: Tassel: Anthocyanin colouration of anther (in middle third of main axis), 8: Tassel: Density of spikelets (in middle third main axis), 9: Tassel: Angle between main axis and lateral branches (in lower third of tassel), 10: Tassel: Attitude of lateral branches (in lower third of tassel) , 11: Ear: time of silk emergence (50% plants), 12: Ear: Anthocyanin colouration of silks (on day of emergence), 13: Leaf: Anthocyanin colouration of sheath (below the ear), 14: Tassel: Length of main axis above lowest side branch, 15: Plant : Height (length up to flag leaf), 16: Plant ear placement, 17: Leaf: width of blade ( leaf of upper ear), 18: Ear: Length without husk, 19: Ear: Diameter without husk, 20: Ear shape, 21: Ear: Number of rows of grains, 22: Ear: Type of grain (in middle third of ear), 23: Ear: colour of top of grains, 24: Ear: Anthocyanin colouration of glumes of cob, 25: Kernel: Row arrangement (middle of ear), 26: Kernel: Poppiness, 27: Kernel: sweetness, 28: Kernel: waxiness, 29: Kernel: Opaqueness, 30: Kernel: shape and 31: Kernel: 1000 kernel weight

**Table S10: ANOVA for different quality parameters of reconstituted and original hybrids**

| **Source of variation** | **df** | **Mean sum of square** | | | | | | | | |
| --- | --- | --- | --- | --- | --- | --- | --- | --- | --- | --- |
|  |  | **AT** | **GT** | **DT** | **TT** | **AT/GT** | **AT/TT** | **ProA** | **Lysine** | **Tryptophan** |
| Replicates | 1 | 0.05 | 3.77 | 0.06 | 148.15** | 0.001 | 0.013** | 0.048 | 0.006* | 0.000 |
| Locations | 2 | 6.17 | 0.60 | 0.71 | 179.46** | 0.012 | 0.006* | 1.184** | 0.000 | 0.00004 |
| Crosses | 16 | 106.68** | 187.97** | 45.31** | 365.06** | 0.486** | 0.035** | 8.984** | 0.004** | 0.00023** |
| Location × Crosses | 32 | 1.87 | 3.53 | 1.06 | 7.61 | 0.013 | 0.001 | 0.721** | 0.001 | 0.00002 |
| Error | 50 | 2.57 | 4.06 | 1.19 | 14.76 | 0.010 | 0.001 | 0.170 | 0.00116 | 0.00003 |
| Total | 101 | 18.89 | 32.96 | 8.12 | 72.57 | 0.087 | 0.007 | 1.760 | 0.00158 | 0.00006 |

* & **: Significance at 5% and 1% level of significance, respectively, df: degree of freedom, AT: α-tocopherol; GT: γ-tocopherol; DT: δ-tocopherol; TT: total tocopherol; proA: provitamin-A

**Table S11: ANOVA for different yield and morphological traits among reconstituted and original hybrids**

| **Source of variation** | **df** | **Mean sum of square** | | | | | | | | | |
| --- | --- | --- | --- | --- | --- | --- | --- | --- | --- | --- | --- |
|  |  | **GY** | **MF** | **FF** | **PH** | **EH** | **EL** | **EG** | **NR** | **NKR** | **100KW** |
| Replicates | 1 | 81543.69 | 0.79 | 0.25 | 108.71 | 86.99 | 2.27 | 0.07 | 3.14* | 36.36* | 0.567 |
| Locations | 2 | 22484548.74** | 1341.54** | 1298.72** | 2029.75** | 3237.53** | 121.07** | 1.38** | 13.03** | 14.40 | 45.08** |
| Crosses | 16 | 2561181.96** | 23.26** | 23.38** | 323.01** | 198.03** | 7.49** | 0.05** | 3.99** | 24.32** | 8.65** |
| Location × Crosses | 32 | 1352059.40** | 5.42* | 6.02** | 148.66** | 76.90** | 2.55* | 0.03 | 0.81 | 27.12** | 5.328 |
| Error B | 50 | 244902.35 | 2.87 | 2.77 | 34.46 | 24.14 | 1.49 | 0.02 | 0.71 | 5.48 | 2.83 |
| Total | 101 | 1401391.06 | 33.40 | 32.70 | 156.60 | 132.66 | 5.15 | 0.05 | 1.53 | 15.80 | 5.36 |

* & **: Significance at 5% and 1% level of significance, respectively, df: degree of freedom, GY: grain yield; MF: days to 50% male flowering; FF: days to 50% female flowering; PH: plant height; EH: ear height; EL: ear length; EG: ear girth; NR: Number of rows; NKR: number of kernels per row; HKW: 100 kernel weight

**Table S12: Mean performance of *ZmVTE4* introgressed hybrids and their original hybrids for yield and yield attributes**

| **Genotype** | **GY**  **(kg/ha)** | **MF**  **(Days)** | **FF**  **(Days)** | **PH**  **(cm)** | **EH**  **(cm)** | **EL**  **(cm)** | **EG**  **(cm)** | **NR**  **(No.)** | **NKR**  **(No.)** | **100KW**  **(gram)** |
| --- | --- | --- | --- | --- | --- | --- | --- | --- | --- | --- |
| **HQPM1-VA** | 8207 | 55 | 57 | 191.1 | 92.8 | 16.2 | 4.4 | 13.8 | 33.8 | 28.3 |
| HQPM1-VA+VE-I | 8743 | 58 | 60 | 206.2 | 102.9 | 18.7 | 4.2 | 13.9 | 37.8 | 25.5 |
| HQPM1-VA+VE-II | 8502 | 57 | 59 | 206.4 | 101.1 | 18.0 | 4.1 | 14.1 | 33.8 | 27.0 |
| HQPM1-VA+VE-III | 8344 | 56 | 59 | 188.2 | 94.0 | 19.6 | 4.2 | 14.0 | 36.4 | 26.3 |
| **Mean** | 8530 | 57 | 59 | 200.3 | 99.3 | 18.8 | 4.2 | 14.0 | 36.0 | 26.3 |
|  | | | | | | | | | | |
| **HQPM4-VA** | 7084 | 55 | 58 | 199.4 | 108.3 | 17.6 | 4.1 | 13.2 | 34.0 | 27.7 |
| HQPM4-VA+VE-I | 7694 | 55 | 58 | 200.1 | 100.6 | 18.1 | 4.1 | 12.6 | 30.2 | 29.0 |
| HQPM4-VA+VE-II | 7365 | 54 | 57 | 183.8 | 99.5 | 17.5 | 4.1 | 13.1 | 30.8 | 27.1 |
| HQPM4-VA+VE-III | 7036 | 56 | 59 | 201.5 | 107.7 | 17.6 | 4.2 | 13.2 | 34.5 | 27.7 |
| **Mean** | 7365 | 55 | 58 | 195.1 | 102.6 | 17.7 | 4.1 | 12.9 | 31.8 | 27.9 |
|  | | | | | | | | | | |
| **HQPM5-PVA** | 8546 | 56 | 59 | 200.1 | 105.5 | 16.5 | 4.3 | 13.9 | 33.2 | 26.9 |
| HQPM5-VA+VE-I | 8326 | 57 | 60 | 206.0 | 115.5 | 17.8 | 4.0 | 12.9 | 36.3 | 27.4 |
| HQPM5-VA+VE-II | 8098 | 59 | 61 | 210.7 | 106.7 | 18.0 | 4.2 | 13.0 | 36.8 | 27.6 |
| HQPM5-VA+VE-III | 8573 | 56 | 59 | 202.7 | 104.6 | 18.8 | 4.1 | 12.9 | 36.7 | 30.4 |
| **Mean** | 8332 | 57 | 60 | 206.5 | 108.9 | 18.2 | 4.1 | 12.9 | 36.6 | 28.5 |
|  | | | | | | | | | | |
| **HQPM7-VA** | 7548 | 55 | 58 | 194.9 | 95.9 | 17.6 | 4.2 | 13.3 | 34.8 | 28.7 |
| HQPM7-VA+VE-I | 7897 | 55 | 57 | 206.9 | 106.1 | 20.4 | 4.2 | 13.2 | 34.3 | 28.0 |
| HQPM7-VA+VE-II | 7712 | 56 | 58 | 200.2 | 99.7 | 17.1 | 4.2 | 13.7 | 34.1 | 27.1 |
| HQPM7-VA+VE-III | 8080 | 55 | 58 | 199.4 | 100.1 | 17.2 | 4.2 | 14.0 | 33.8 | 28.2 |
| **Mean** | 7896 | 55 | 58 | 202.1 | 101.9 | 18.2 | 4.2 | 13.6 | 34.0 | 27.8 |
|  | | | | | | | | | | |
| **PVQ9I** (Commercial check) | 6337 | 49 | 52 | 191.5 | 98.4 | 16.1 | 4.3 | 16.2 | 33.2 | 25.7 |
| **SE_d_** | 285.72 | 0.98 | 0.96 | 3.39 | 2.84 | 0.70 | 0.08 | 0.49 | 1.35 | 0.97 |

GY: grain yield; MF: days to 50% male flowering; FF: days to 50% female flowering; PH: plant height; EH: ear height; EL: ear length; EG: ear girth; NR: Number of rows; NKR: number of kernels per row; HKW: 100 kernel weight

**Table S13: DUS characteristics of *vte4-*introgressed reconstituted hybrids and their respective original hybrids**

| **Genotype** | **1** | **2** | **3** | **4** | **5** | **6** | **7** | **8** | **9** | **10** | **11** | **12** | **13** | **14** | **15** | **16** |
| --- | --- | --- | --- | --- | --- | --- | --- | --- | --- | --- | --- | --- | --- | --- | --- | --- |
| **HQPM1-VA** | Small | Drooping | Absent | Late | Absent | Absent | Present | Dense | Narrow | Straight | Late | Present | Present | Long | Long | Medium |
| HQPM1-VA+VE-I | Small | Drooping | Absent | Late | Absent | Present | Present | Dense | Narrow | Straight | Late | Present | Present | Long | Long | Medium |
| HQPM1-VA+VE-II | Small | Drooping | Absent | Late | Absent | Present | Present | Dense | Narrow | Straight | Late | Present | Present | Long | Long | Medium |
| HQPM1-VA+VE-III | Small | Drooping | Absent | Late | Absent | Present | Present | Dense | Narrow | Straight | Late | Present | Present | Long | Long | Medium |
|  | | | | | | | | | | | | | | | | |
| **HQPM4-VA** | Small | Drooping | Absent | Late | Absent | Present | Absent | Dense | Narrow | Straight | Late | Present | Present | Long | Long | Medium |
| HQPM4-VA+VE-I | Small | Drooping | Absent | Late | Absent | Absent | Present | Dense | Narrow | Straight | Late | Present | Present | Long | Long | Medium |
| HQPM4-VA+VE-II | Small | Drooping | Absent | Late | Absent | Present | Present | Dense | Narrow | Straight | Late | Present | Present | Long | Long | Medium |
| HQPM4-VA+VE-III | Small | Drooping | Absent | Late | Absent | Present | Present | Dense | Narrow | Straight | Late | Present | Present | Long | Long | Medium |
|  | | | | | | | | | | | | | | | | |
| **HQPM5-VA** | Wide | Drooping | Present | Medium | Absent | Present | Present | Dense | Narrow | Straight | Medium | Present | Present | Long | Long | Medium |
| HQPM5-VA+VE-I | Wide | Drooping | Absent | Medium | Absent | Present | Present | Dense | Narrow | Straight | Medium | Present | Present | Long | Long | Medium |
| HQPM5-VA+VE-II | Wide | Drooping | Absent | Medium | Absent | Present | Present | Dense | Narrow | Straight | Medium | Present | Present | Long | Long | Medium |
| HQPM5-VA+VE-III | Wide | Drooping | Absent | Medium | Absent | Present | Present | Dense | Narrow | Straight | Medium | Present | Present | Long | Long | Medium |
|  | | | | | | | | | | | | | | | | |
| **HQPM7-VA** | Wide | Drooping | Present | Medium | Absent | Present | Present | Dense | Narrow | Straight | Medium | Present | Present | Long | Long | Medium |
| HQPM7-VA+VE-I | Wide | Drooping | Absent | Medium | Absent | Present | Present | Dense | Narrow | Straight | Medium | Present | Present | Long | Long | Medium |
| HQPM7-VA+VE-II | Wide | Drooping | Absent | Medium | Absent | Present | Present | Dense | Narrow | Straight | Medium | Present | Present | Long | Long | Medium |
| HQPM7-VA+VE-III | Wide | Drooping | Absent | Medium | Absent | Present | Present | Dense | Narrow | Straight | Medium | Present | Present | Long | Long | Medium |

| **Genotype** | **17** | **18** | **19** | **20** | **21** | **22** | **23** | **24** | **25** | **26** | **27** | **28** | **29** | **30** | **31** |
| --- | --- | --- | --- | --- | --- | --- | --- | --- | --- | --- | --- | --- | --- | --- | --- |
| **HQPM1-VA** | Medium | Long | Medium | Conico-cylindrical | Medium | Semi-flint | Yellow | Absent | Straight | Absent | Absent | Absent | Present | Round | Large |
| HQPM1-VA+VE-I | Broad | Long | Medium | Conico-cylindrical | Medium | Semi-flint | Yellow | Absent | Straight | Absent | Absent | Absent | Present | Round | Large |
| HQPM1-VA+VE-II | Broad | Long | Medium | Conico-cylindrical | Medium | Semi-flint | Yellow | Absent | Straight | Absent | Absent | Absent | Present | Round | Large |
| HQPM1-VA+VE-III | Broad | Long | Medium | Conico-cylindrical | Medium | Semi-flint | Yellow | Absent | Straight | Absent | Absent | Absent | Present | Round | Large |
|  | | | | | | | | | | | | | | | |
| **HQPM4-VA** | Broad | Long | Medium | Conico-cylindrical | Medium | Flint | Orange | Absent | Straight | Absent | Absent | Absent | Present | Round | Large |
| HQPM4-VA+VE-I | Broad | Long | Medium | Conico-cylindrical | Medium | Flint | Yellow orange | Absent | Straight | Absent | Absent | Absent | Present | Round | Large |
| HQPM4-VA+VE-II | Broad | Long | Medium | Conico-cylindrical | Medium | Flint | Orange | Absent | Straight | Absent | Absent | Absent | Present | Round | Large |
| HQPM4-VA+VE-III | Broad | Long | Medium | Conico-cylindrical | Medium | Flint | Orange | Absent | Straight | Absent | Absent | Absent | Present | Round | Large |
|  | | | | | | | | | | | | | | | |
| **HQPM5-VA** | Medium | Long | Medium | Conico-cylindrical | Many | Semi-flint | Orange | Absent | Straight | Absent | Absent | Absent | Present | Round | Large |
| HQPM5-VA+VE-I | Medium | Long | Medium | Conico-cylindrical | Many | Flint | Orange | Absent | Straight | Absent | Absent | Absent | Present | Round | Large |
| HQPM5-VA+VE-II | Broad | Long | Medium | Conico-cylindrical | Many | Flint | Orange | Absent | Straight | Absent | Absent | Absent | Present | Round | Large |
| HQPM5-VA+VE-III | Broad | Long | Medium | Conico-cylindrical | Many | Flint | Orange | Absent | Straight | Absent | Absent | Absent | Present | Round | Large |
|  | | | | | | | | | | | | | | | |
| **HQPM7-VA** | Medium | Long | Medium | Conico-cylindrical | Many | Semi-flint | Orange | Absent | Straight | Absent | Absent | Absent | Present | Round | Large |
| HQPM7-VA+VE-I | Broad | Long | Medium | Conico-cylindrical | Many | Semi-flint | Orange | Absent | Straight | Absent | Absent | Absent | Present | Round | Large |
| HQPM7-VA+VE-II | Broad | Long | Medium | Conico-cylindrical | Many | Semi-flint | Orange | Absent | Straight | Absent | Absent | Absent | Present | Round | Large |
| HQPM7-VA+VE-III | Medium | Long | Medium | Conico-cylindrical | Many | Semi-flint | Yellow orange | Absent | Straight | Absent | Absent | Absent | Present | Round | Large |

1: Leaf: Angle between blade and stem (on leaf just above upper ear), 2: Leaf: Attitude of blade (on leaf just above upper ear), 3: Stem: Anthocyanin colouration of brace root, 4: Time of anthesis (on middle third of main axis 50 % of plants), 5: Tassel: Anthocyanin colouration at base of glume (in middle third of main axis), 6: Tassel: Anthocyanin colouration excluding base (in middle third of main axis), 7: Tassel: Anthocyanin colouration of anther (in middle third of main axis), 8: Tassel: Density of spikelets (in middle third main axis), 9: Tassel: Angle between main axis and lateral branches (in lower third of tassel), 10: Tassel: Attitude of lateral branches (in lower third of tassel) , 11: Ear: time of silk emergence (50% plants), 12: Ear: Anthocyanin colouration of silks (on day of emergence), 13: Leaf: Anthocyanin colouration of sheath (below the ear), 14: Tassel: Length of main axis above lowest side branch, 15: Plant : Height (length up to flag leaf), 16: Plant ear placement, 17: Leaf: width of blade ( leaf of upper ear), 18: Ear: Length without husk, 19: Ear: Diameter without husk, 20: Ear shape, 21: Ear: Number of rows of grains, 22: Ear: Type of grain (in middle third of ear), 23: Ear: colour of top of grains, 24: Ear: Anthocyanin colouration of glumes of cob, 25: Kernel: Row arrangement (middle of ear), 26: Kernel: Poppiness, 27: Kernel: sweetness, 28: Kernel: waxiness, 29: Kernel: Opaqueness, 30: Kernel: shape and 31: Kernel: 1000 kernel weigh

**1 2 3 4 5 6 7 8 9 10 11 12 L 13 14 15 16 17 18 19 20 21 22 23 24**


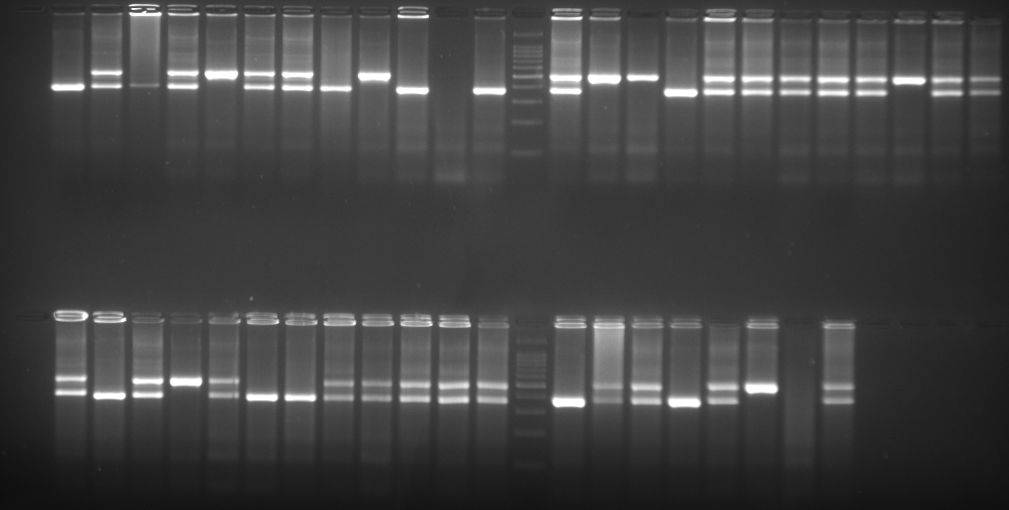


**Figure S1: Foreground selection for *vte4* (InDel118) alleles in BC_2_F_2_ generation of the RP × DP** (L-100bp ladder)


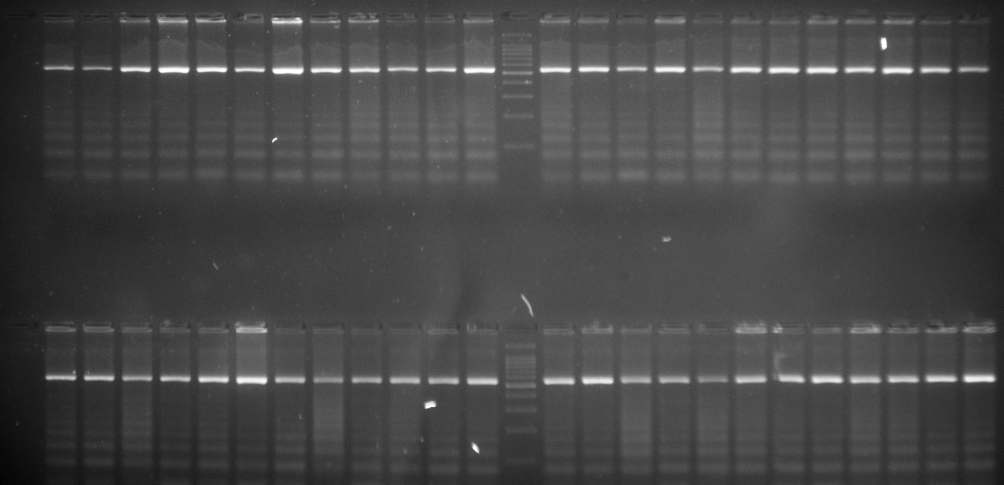


**DP RP 1 2 3 4 5 6 7 8 9 10 11 L 12 13 14 15 16 17 18 19 20 21 22 23 1 2 3 4 5 6 7 8 9 10 11 12 L 13 14 15 16 17 18 19 20 21 22 23 24**

**Figure S2: Foreground selection for *crtRB1-3’TE* allele in BC_2_F_2_ of the RP × DP** (RP: Recurrent parent; DP: Donor parent; L: 100bp ladder)


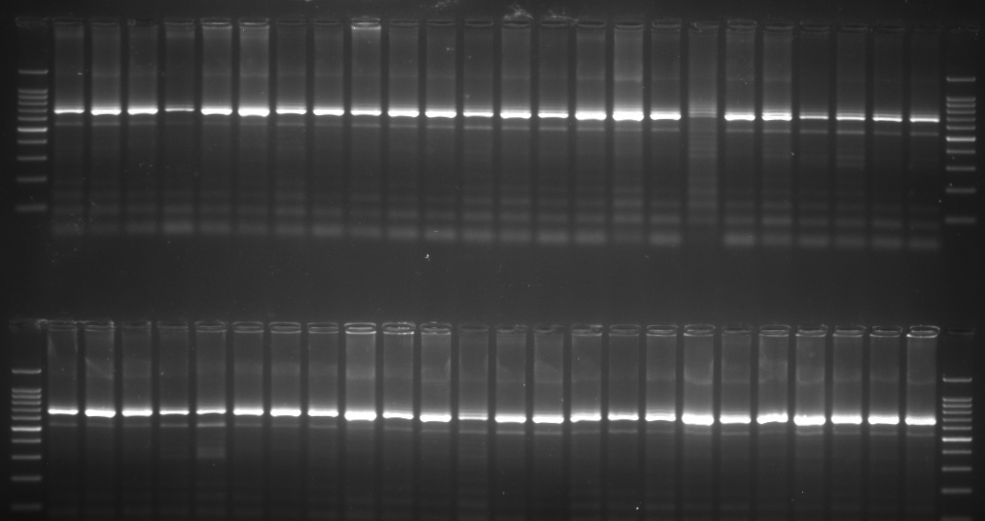


**L DP RP 1 2 3 4 5 6 7 8 9 10 11 12 13 14 15 16 17 18 19 20 21 22 L**

**Figure S3: Foreground selection for *lcyE-5’TE* alleles in BC_2_F_2_ of the RP × DP** (RP: Recurrent parent; DP: Donor parent; L: 100bp ladder)


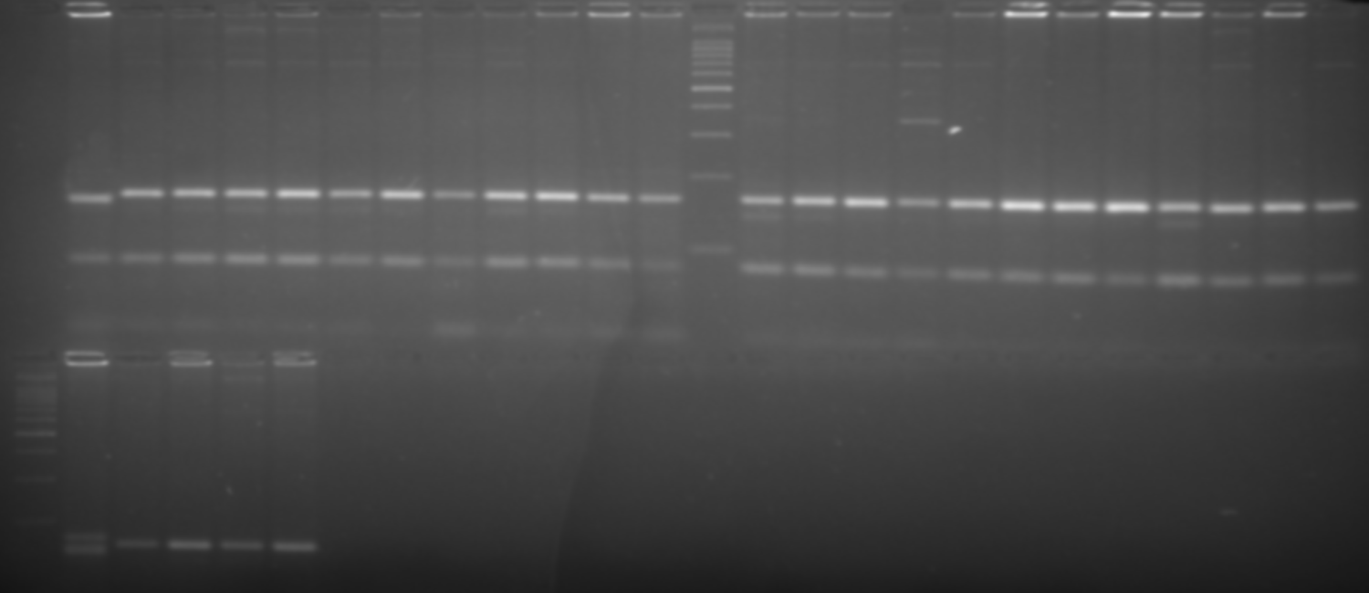


**DP RP 1 2 3 4 5 6 7 8 9 10 L 11 12 13 14 15 16 17 18 19 20 21 22**

**Figure S4: Foreground selection for *opaque2* (*phi057*) alleles in BC_2_F_2_ of the RP × DP** (RP: Recurrent parent; DP: Donor parent; L: 100bp ladder)


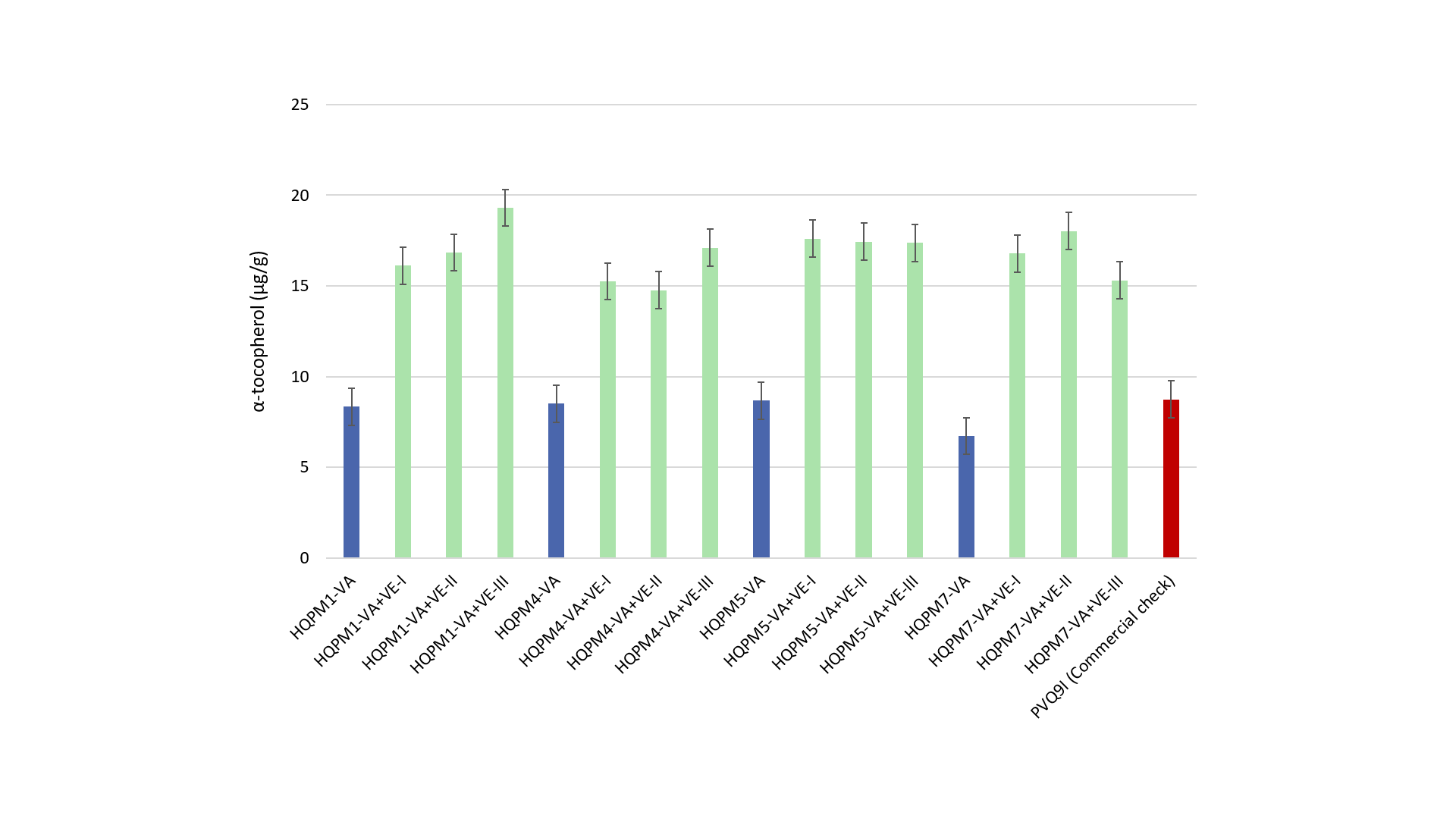


Figure S5: Concentration of α-tocopherol in the reconstituted (green bars), original (blue bars) hybrids, and commercial check (red bars)


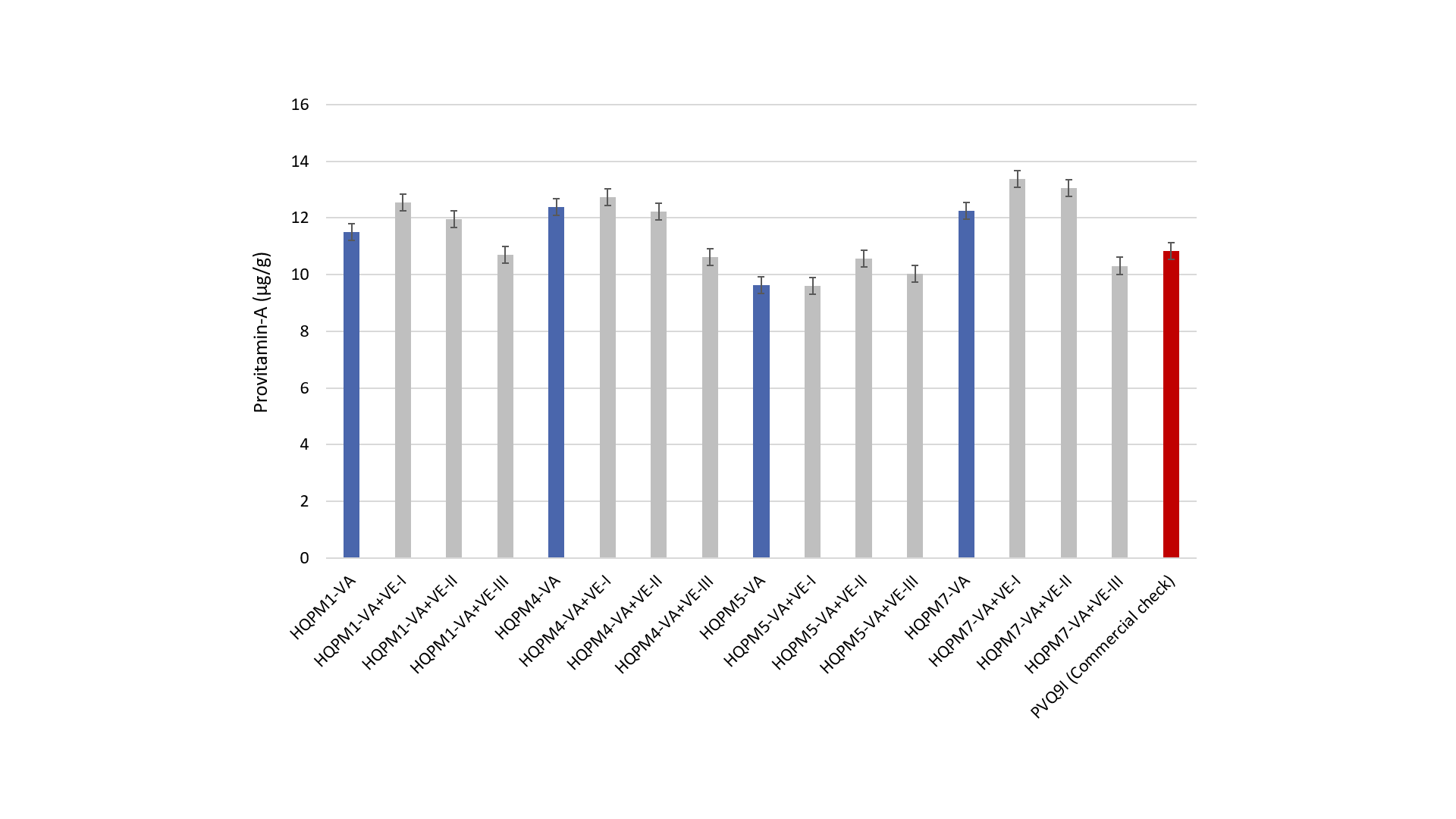


Figure S6: Concentration of provitamin-A in the reconstituted (grey bars), original (blue bars) hybrids, and commercial check (red bars)


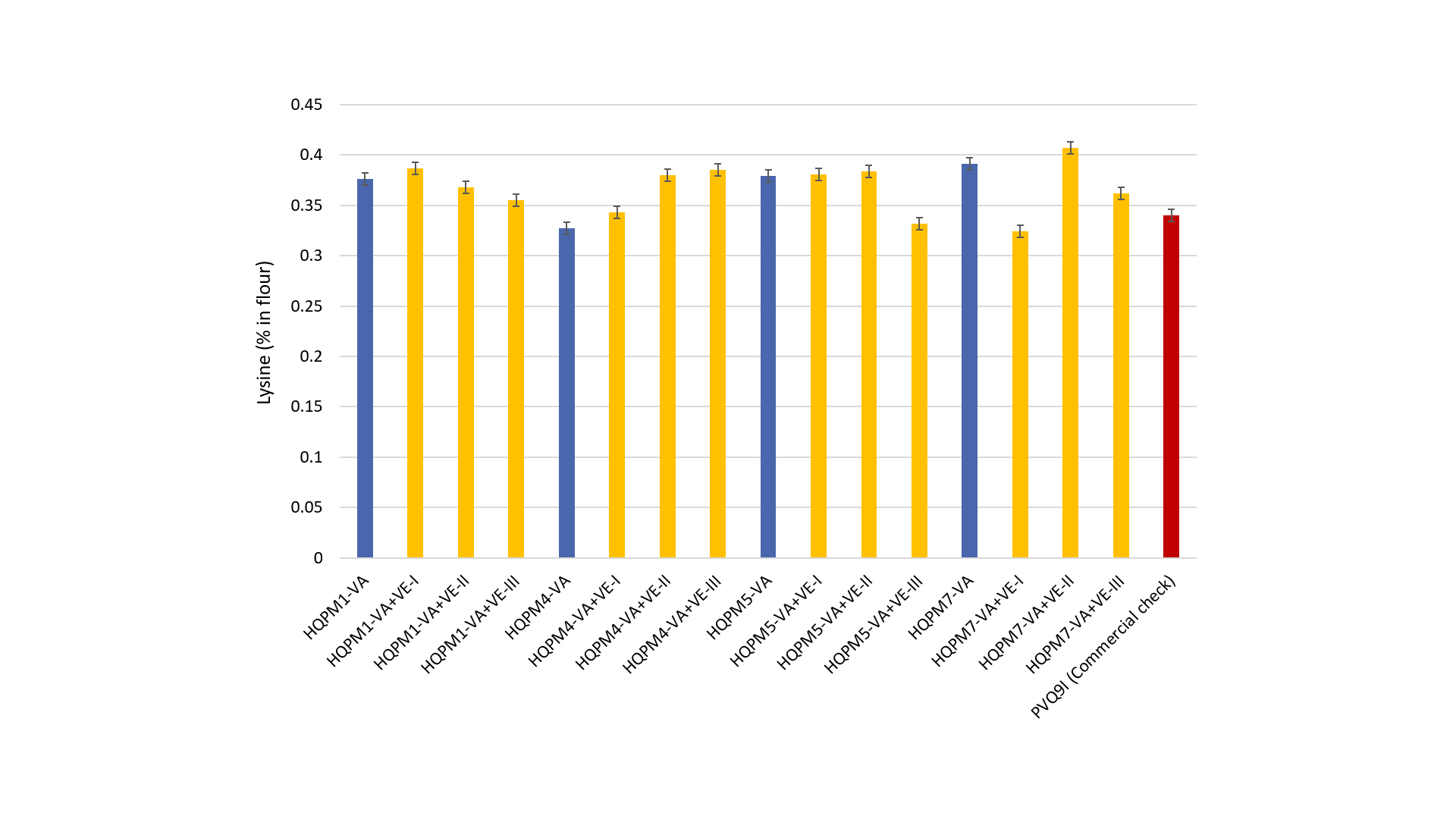


Figure S7: Concentration of lysine in the reconstituted (orange bars), original (blue bars) hybrids, and commercial check (red bars)


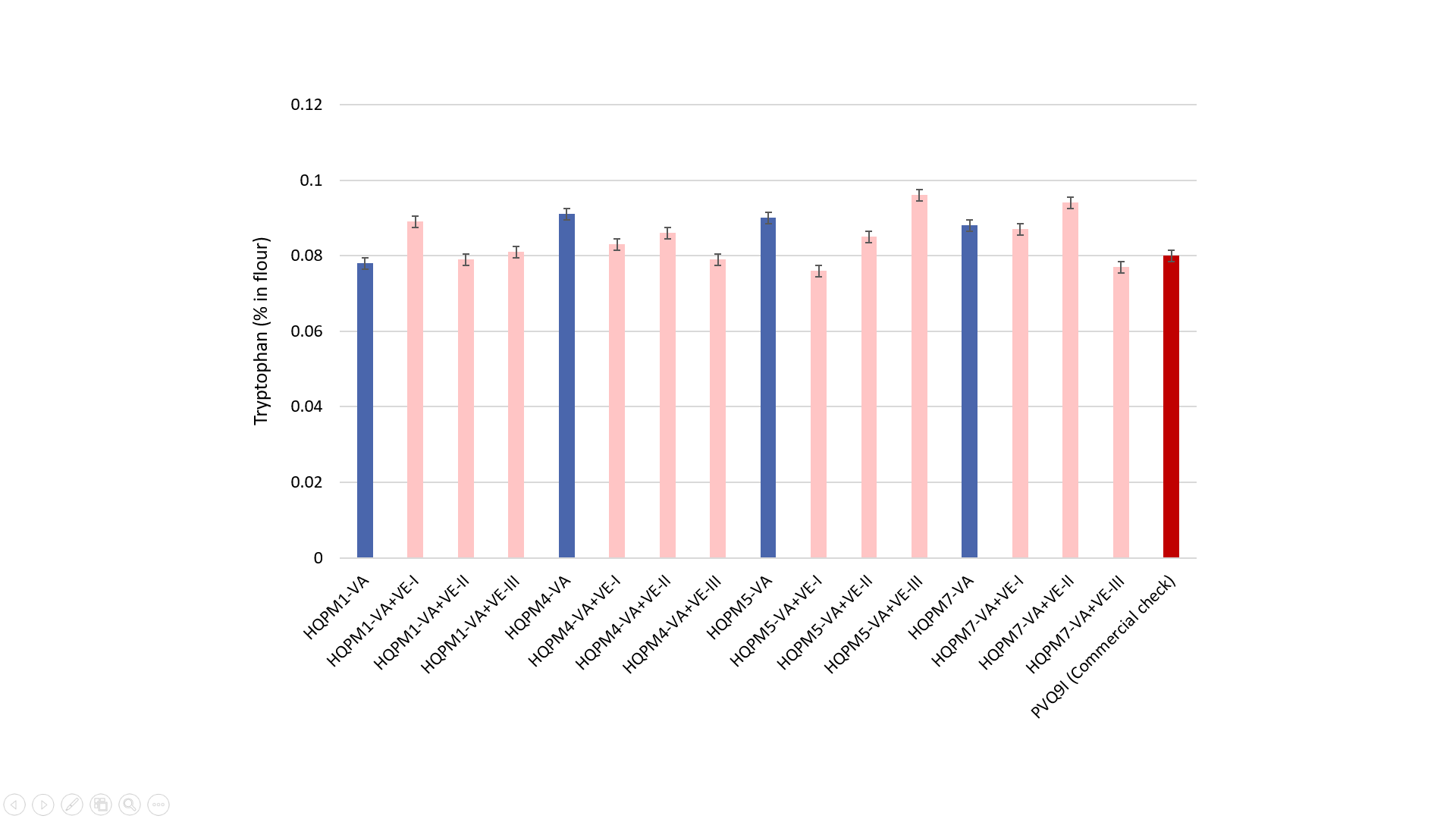


Figure S8: Concentration of tryptophan in the reconstituted (pink), original (blue bars) hybrids, and commercial check (red bars)
